# The Development and Use of AI Chatbots for Health Behavior Change: Scoping Review

**DOI:** 10.2196/79677

**Published:** 2026-01-28

**Authors:** Lingyi Fu, Ryan Burns, Yuhuan Xie, Jincheng Shen, Shandian Zhe, Paul Estabrooks, Yang Bai

**Affiliations:** 1 Department of Health and Kinesiology College of Health University of Utah Salt Lake City, UT United States; 2 Department of Internal Medicine School of Medicine University of Utah Salt Lake City, UT United States; 3 Kahlert School of Computing University of Utah Salt Lake City, UT United States

**Keywords:** conversational agent, machine learning, health behavior change, physical activity, diet, sleep

## Abstract

**Background:**

Artificial intelligence (AI) chatbots are technologies that facilitate human-computer interaction through communication in a natural language format. By increasing cost-effectiveness, interaction, autonomy, personalization, and support, mobile health interventions can benefit health behavior change and make it more natural and intuitive.

**Objective:**

This study aimed to provide an up-to-date and practical overview of how text-based AI chatbots are designed, developed, and evaluated across 8 health behaviors, including their roles, theoretical foundations, health behavior change techniques, technology development workflow, and performance validation framework.

**Methods:**

In accordance with the PRISMA-ScR (Preferred Reporting Items for Systematic Reviews and Meta-Analyses extension for Scoping Reviews) framework, relevant studies published before March 2024 were identified from 9 bibliographic databases (ie, PubMed, CINAHL, MEDLINE, Embase, Web of Science, Scopus, APA PsycINFO, IEEE Xplore, and ACM Digital Library). Two stages (ie, title and abstract screening followed by full-text screening) were conducted to screen the eligibility of the papers via Covidence software. Finally, we extracted the data via Microsoft Excel software and used a narrative approach, content analysis, and evidence map to synthesize the reported results.

**Results:**

Our systematic search initially identified 10,508 publications, 43 of which met our inclusion criteria. AI chatbots primarily served 2 main roles: routine coach (27/43, 62.79%) and on-demand assistant (12/43, 27.91%), while 4 studies (4/43, 9.30%) integrated both roles. Frameworks like cognitive behavioral therapy (13/24, 54.17%) and behavior change techniques, such as goal setting, feedback and monitoring, and social support, guided the development of theory-driven AI chatbots. Noncode platforms (eg, Google Dialogflow and IBM Watson) integrated with social messaging platforms (eg, Facebook Messenger) were commonly used to develop AI chatbots (23/43, 53.49%). AI chatbots have been evaluated across 4 domains: technical performance (17/43, 39.53%), usability (17/43, 39.53%), engagement (37/43, 86.05%), and health behavior change (33/43, 76.74%). Evidence for health behavior changes remains exploratory but promising. Among 33 studies with 120 comparisons, 81.67% (98/120) showed positive outcomes, though only 35.83% (43/120) had moderate or larger effects (Hedges g or odds ratio or Cohen d>0.5). Most involved nonclinical (36/43, 83.72%) and adults (23/43, 53.49%), and a few were randomized controlled trials (14/43, 32.56%). Benefits were mainly seen in physical activity, smoking cessation, stress management, and diet, with limited evidence for other behaviors. Findings were inconsistent regarding the influence of long-term effects, intervention duration, modality, and engagement on health behavior change outcomes.

**Conclusions:**

The exploratory synthesis provides a roadmap for developing and evaluating AI chatbots in health behavior change, highlighting the need for further research on cost, implementation outcomes, and underexplored behaviors such as sleep, weight management, sedentary behavior, and alcohol use.

## Introduction

Health-related behavior (hereafter referred to as “health behaviors”) is essential for improving population health worldwide [1]. Engaging in health-promoting behaviors, such as having a healthy diet, getting adequate physical activity (PA) and sleep, and avoiding health-risk behaviors, such as smoking, can substantially reduce chronic disease and all-cause mortality risk [1,2] as well as benefit mental health [3]. Despite these benefits, unhealthy behaviors remain a significant public health concern and place a substantial burden on health care systems [4]. Health coaching is one suggested intervention for promoting health behavior changes [5,6]. Health coaching is defined as “the practice of health education and health promotion within a coaching context, to increase the well-being of individuals and to facilitate the achievement of their health-related goals” [7]. Most health coaching interventions are delivered by humans in various ways, such as face-to-face, telephonically, or via email [5]. Effective communication between coaches and users can support user-centered care and shared decision-making [8]. However, human health coaching interventions are limited in their ability to reach everyone in need of support because of a lack of coaching practitioners, resources in resource-limited communities, and barriers for individuals in accessing coaching support, such as low income [9]. From the implementer’s perspective, human health coaching often lacks consistent data collection, continuous monitoring, scalability, and long-term sustainability [10]. Therefore, finding resource-efficient, cost-effective, and easily implementable strategies to promote health behavior change can be helpful to alleviate an already burdened health care system.

A chatbot is a computer program designed to respond to conversational or informational replies with verbal (audio-based chatbot) or written (text-based chatbot) messages from users [11]. This technology can be another type of resource for delivering health coaching, complemented by traditional human health coaching [9]. Chatbots can be developed with and without artificial intelligence (AI) algorithms. Most prior chatbots for health behavior change were developed without AI algorithms (“Non-AI chatbots”) used in template-, rule-, or retrieval-based dialogue systems [12]. These chatbots responded to users by selecting from a predefined list, allowing for a high degree of researcher control but lacking the conversational flexibility and personalization typically offered by human coaches [9,13]. Recently, with advances in AI in the health care field, some chatbots have been developed using AI algorithms [14], such as reinforcement learning, deep neural networks, and random forest. These AI chatbots can communicate with users in natural language [15-20], offering personalized support, multimodal reasoning, and greater conversational flexibility and intuitiveness [21]. In particular, AI chatbots can support health behavior change in various ways. Research has shown that motivational interviewing (MI)–based AI chatbot tends to be perceived as more empathetic and trustworthy than the directed intervention, and it significantly raises the participants’ self-efficacy to overcome barriers and positively impacts intrinsic motivation and PA levels [22]. Additionally, AI chatbots can also help alleviate stress by enhancing perceived supportiveness through the provision of emotional support [23]. Although the benefits are numerous, several concerns remain, including privacy and security [16]; limited empathy, affect, and emotional support [24]; low engagement; and challenges in monitoring intervention fidelity. Therefore, it is important to provide evidence to address these concerns and ensure the efficacy and safety of chatbot interventions.

Previous review studies have reported varying evidence on the application of AI chatbots for health behavior change. For example, a meta-analysis demonstrated strong efficacy of chatbot-based interventions in increasing physical activity, fruit and vegetable consumption, and sleep duration and quality. The analysis also showed that the effects varied by intervention duration, intervention modality (chatbot-only vs multicomponent interventions), and chatbot characteristics (text-based vs audio-based and AI-driven vs non–AI-driven chatbots) [12]. Other systematic reviews have summarized the outcome in addition to efficacy, including engagement, acceptability, satisfaction, and safety [25], as well as feasibility, usability, and intervention characteristics [10]. However, there remains a lack of research exploring key topics, such as the role of AI chatbots in behavior change, the health behavior change techniques (BCTs) adopted by AI chatbots, comprehensive technology frameworks for chatbot development, and frameworks for performance validation. Therefore, to address this gap and complement existing review studies [10,12,25], it is necessary to conduct a scoping review to provide a comprehensive overview of existing research for both scholars and practitioners in this field. This scoping review aimed to provide an up-to-date and practical examination of the design (ie, roles, theories, and health BCTs), development (technology workflow), and use (ie, performance validation tool) of text-based AI chatbots for 8 health behaviors, including PA, diet, sleep, weight management, sedentary, stress management, smoking cessation, and alcohol. In particular, 4 specific research questions were proposed based on the indications from the scoping reviews [26]:

- Question 1: What are the most commonly targeted health behaviors in text-based AI chatbots?
- Question 2: What roles, theoretical foundations, and BCTs are applied in text-based AI chatbots, supporting health behavior change interventions?
- Question 3: What technologies are used to develop text-based AI chatbots for health behavior change?
- Question 4: How to validate text-based AI chatbot performance in health behavior change?
  Question 4.1: What measures are used to assess technical performance, usability, engagement, and cost?
  Question 4.2: What are the health behavior change outcomes?

## Methods

### Protocol and Registration

The scoping review process was designed following the PRISMA-ScR (Preferred Reporting Items for Systematic Reviews and Meta-Analysis extension for Scoping Reviews) framework [27]. The PRISMA-ScR checklist is reported in Multimedia Appendix 1. The study has been registered in the Open Science Framework [28], the most common platform for deposit protocols for scoping reviews.

### Search Strategy

#### Search Resources

Relevant studies published before March 2024 were identified through 9 bibliographic databases, including 4 widely used health science databases (ie, PubMed, CINAHL, MEDLINE, and Embase), 2 multidisciplinary databases (ie, Web of Science and Scopus), 1 behavior and social science database (ie, APA PsycINFO), and 2 technology databases (ie, IEEE Xplore and ACM Digital Library). The last search date for each database is March 13, 2024.

#### Search Terms

Our search strategy incorporated terms related to both the intervention (AI chatbots) and the outcomes (health behaviors; Table S1 in Multimedia Appendix 2). Given the 6 pillars of lifestyle medicine [29], this study focused on 8 health behaviors, including PA, diet, sleep, weight management, sedentary behavior, stress management, smoking cessation, and alcohol. We identified synonyms for AI chatbots and various health behaviors by searching for relevant terms in dictionaries and references. We subsequently generated search syntaxes adapted to the specific requirements of each database. The example of search syntax for PubMed is as follows: “(((lifestyle*[tiab] OR tobac*[tiab] OR cigarette*[tiab] OR cigar* [tiab] OR vap*[tiab] OR smok*[tiab] OR nico*[tiab] OR sleep*[tiab] OR bedtime[tiab] OR nap[tiab] OR insomnia[tiab] OR physical activ*[tiab] OR sport*[tiab] OR exercise*[tiab] OR diet*[tiab] OR nutriti*[tiab] OR eating [tiab] OR food*[tiab] OR appetite*[tiab] OR *weight*[tiab] OR obes*[tiab] OR sedentar*[tiab] OR screen time [tiab] OR stress* [tiab]) AND (Chatbot*[tiab] OR chat-bot*[tiab] OR chat bot*[tiab] OR chat robot*[tiab] OR virtual robot*[tiab] OR voice-bot[tiab] OR social bot*[tiab] OR social robot*[tiab] OR infobot*[tiab] OR health bot*[tiab] OR smartbot*[tiab] OR conversational bot*[tiab] OR artificial intelligence chatbot*[tiab] OR Ai agent*[tiab] OR conversational agent*[tiab] OR dialogue agent*[tiab] OR dialog agent* [tiab] OR interactive agent*[tiab] OR virtual agent*[tiab] OR automated agent*[tiab] OR relational agent*[tiab] OR AI assist*[tiab] OR conversational assistant*[tiab] OR digital assist*[tiab] OR intelligent assist*[tiab] OR virtual assist*[tiab] OR smart assist*[tiab] OR voice assist*[tiab] OR speech assist*[tiab] OR virtual health assist*[tiab] OR dialogue agent*[tiab] OR dialog agent*[tiab] OR AI advisor*[tiab] OR virtual advisor*[tiab] OR animated advisor*[tiab] OR smart advisor*[tiab] OR AI avatar*[tiab] OR virtual avatar*[tiab] OR animated avatar*[tiab] OR smart avatar*[tiab] OR AI coach*[tiab] OR virtual coach*[tiab] OR smart coach*[tiab] OR animated coach*[tiab] OR artificial conversation entit*[tiab] OR Assistance technolog*[tiab] OR conversational AI[tiab] OR conversational interface*[tiab] OR conversational system*[tiab] OR Dialog system*[tiab] OR dialogue system*[tiab] OR natural language interface*[tiab] OR automated conversation[tiab] OR virtual conversation[tiab] OR chatGPT[tiab])) AND (eng[la])) NOT (Systematic review[pt] OR meta-analysis[pt] OR review[pt]).” Finally, the metadata of the identified papers were imported into the Covidence platform to eliminate duplication and screening.

### Study Eligibility Criteria

Table S2 in Multimedia Appendix 2 outlines the study eligibility criteria for the study and publication characteristics. The study characteristics were designed based on the PICOS framework, including population, intervention, comparison, outcome, and study type [30]. The publication characteristics included publication date, language, and publication status (eg, full online).

### Study Selection

The selection process had 2 stages: title and abstract screening, followed by full-text screening. Both were conducted independently by 2 reviewers (LF and YX), with conflicts resolved by a third reviewer (YB). We used Cohen κ metric to evaluate interrater agreement [31]. The reviewers achieved substantial agreement in stage 1, with a κ measure of 0.83, which is greater than the cutoff point of 0.81 [31]. The screening process and calculation of Cohen κ value were performed via Covidence software.

### Study Quality Assessment

Given the diversity of study designs, we used the Mixed Methods Appraisal Tool (MMAT) to assess methodological quality [32] (Table S3 in Multimedia Appendix 2 [16-20,23,24,33-68]). The MMAT is a 21-item checklist covering 5 research designs: qualitative, quantitative randomized controlled trials (RCTs), quantitative nonrandomized studies, quantitative descriptive studies, and mixed methods studies. Interrater reliability for the MMAT has been reported to range from moderate to perfect [69]. Two reviewers (LF and YX) independently evaluated each paper, and any disagreements were resolved through discussion. In general, all studies clearly stated their research questions, and the collected data were sufficient to address them.

Assigning an overall numerical score based on MMAT ratings is discouraged, as a single number cannot capture specific methodological issues [32]. Therefore, we presented detailed ratings for each criterion. All eligible studies were included in this review, regardless of their MMAT ratings, as excluding studies solely on the basis of low methodological quality is not recommended [32,70].

### Data Items

We designed the initial elements of the charting form based on the research questions and the mobile health evidence reporting and assessment checklist [71]. The items were primarily developed by 1 investigator (LF) and subsequently verified by another investigator (YB). The items were refined throughout the process, resulting in a final set of 22 elements (Table S4 in Multimedia Appendix 2).

### Charting Process and Data Synthesis

Regarding data charting, 1 reviewer (LF) was primarily responsible for data extraction. This process involved 2 stages: the first stage focused on extraction, and the second stage on confirmation and supplementation. Two additional reviewers (Conrad Ma and Shreya Sanghvi) then independently validated the extracted data. Clear instructions for data validation were provided to these reviewers. Any discrepancies were resolved through discussion among the 3 reviewers. Microsoft Excel software was used for data charting processes. Finally, we used a narrative approach, content analysis, and evidence map to synthesize the reported results. In particular, we conducted a deductive coding process to map each chatbot function to the existing BCT Taxonomy (version 1) [72], enabling cross-study comparisons. For the AI chatbot validation framework in health behavior change, we applied a combined deductive-inductive approach to map the measures from each study onto 5 domains (ie, technical performance, health behavior change, usability, cost, and engagement), drawing from the digital health scorecard framework [73] and engagement framework [74].

## Results

### Study Selection

**Figure 1:**
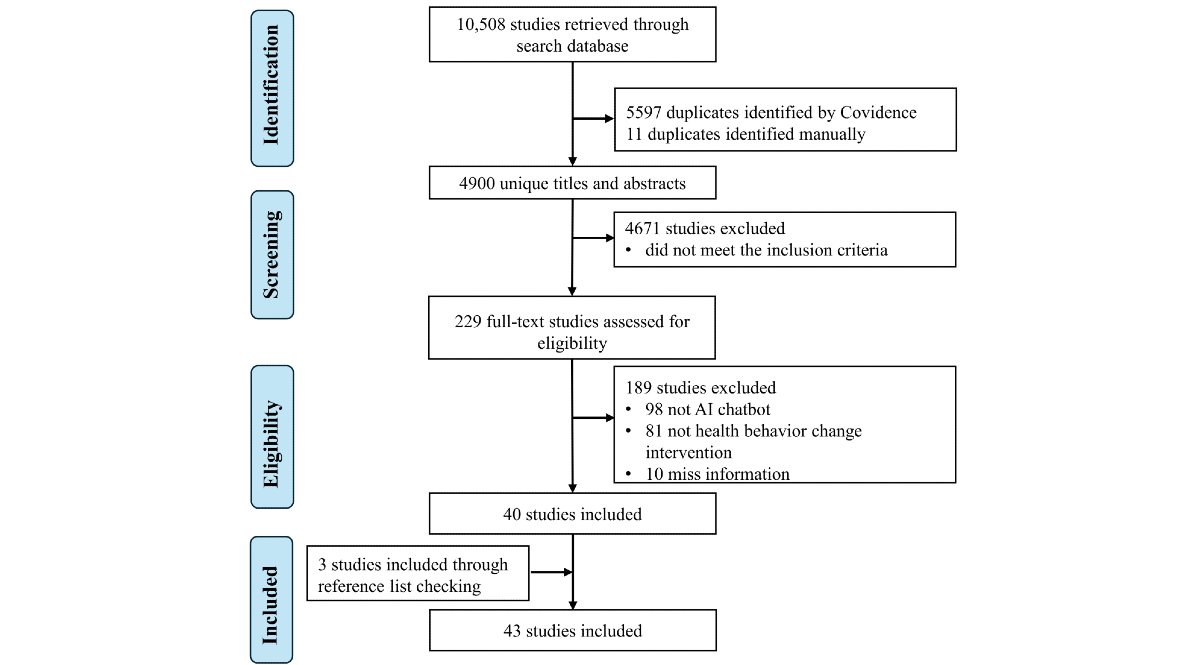
Study selection process. AI: artificial intelligence.

Figure 1 summarizes the process of selecting the studies via the Covidence software (Veritas Health Innovation). A total of 10,508 studies were returned after searching the databases. After duplication removal and title and abstract screening, 229 studies remained. In total, 40 studies remained after the full-text screening phase, with 189 studies removed for the following reasons: not involving an AI chatbot (n=98), not being a health behavior change intervention or implementation study (n=81), or lacking sufficient information (n=10). We included 3 additional studies after forward and backward reference checking, bringing the total to 43 included studies.

### Study Overview

#### Overview

An overview of the studies included is presented in Table S5 in Multimedia Appendix 2 [16-20,23,24,33-68]. We further synthesized the findings across studies. Table S6 in Multimedia Appendix 2 [16-20,23,24,33-68] summarizes the publication characteristics of the studies included in the review. They were published between 2018 and 2024, with most published in 2023 (11/43, 25.58%). The studies were conducted across 15 countries or regions, with most of them conducted in Western countries (32/43, 74.42%), especially the United States (9/32, 28.13%), followed by the Netherlands (5/32, 15.63%), Italy (5/32, 15.63%), and the United Kingdom (5/32, 15.63%). Most research papers were published in journals (33/43, 76.74%), with the largest number published in *JMIR mHealth and uHealth* (6/33, 18.18%).

#### Methodological and Participant Characteristics

Table S7 in Multimedia Appendix 2 [16-20,23,24,33-68] summarizes the methodological and participant characteristics. Studies were classified based on the study types from Hong et al [32], including 14 RCTs, 15 quantitative nonrandomized trials (non-RCTs), 8 quantitative descriptive studies, 2 qualitative studies, and 4 mixed methods studies. The number of participants ranged from 7 [33] to 57,214 [34], with a primary focus on young and middle adulthood (aged 19-64 years; 23/43, 53.49%) based on age categories of Lindemann et al [75]. Most studies targeted nonclinical populations (36/43, 83.72%), such as individuals who are physically inactive, those with unhealthy diets, smokers, substance users, students (middle school, high school, and university), workers, and vulnerable groups, including low-income English- and Spanish-speaking individuals and residents of health professional shortage areas. In total, 7 studies targeted clinical populations (7/43, 16.28%), such as patients with colorectal cancer [35], patients with celiac [36], patients with cardiovascular problem [37], survivors of cancer [38], population with clinical eating disorder [39], as well as children [40] and youths [41] with obesity.

#### Intervention Characteristics

**Figure 2:**
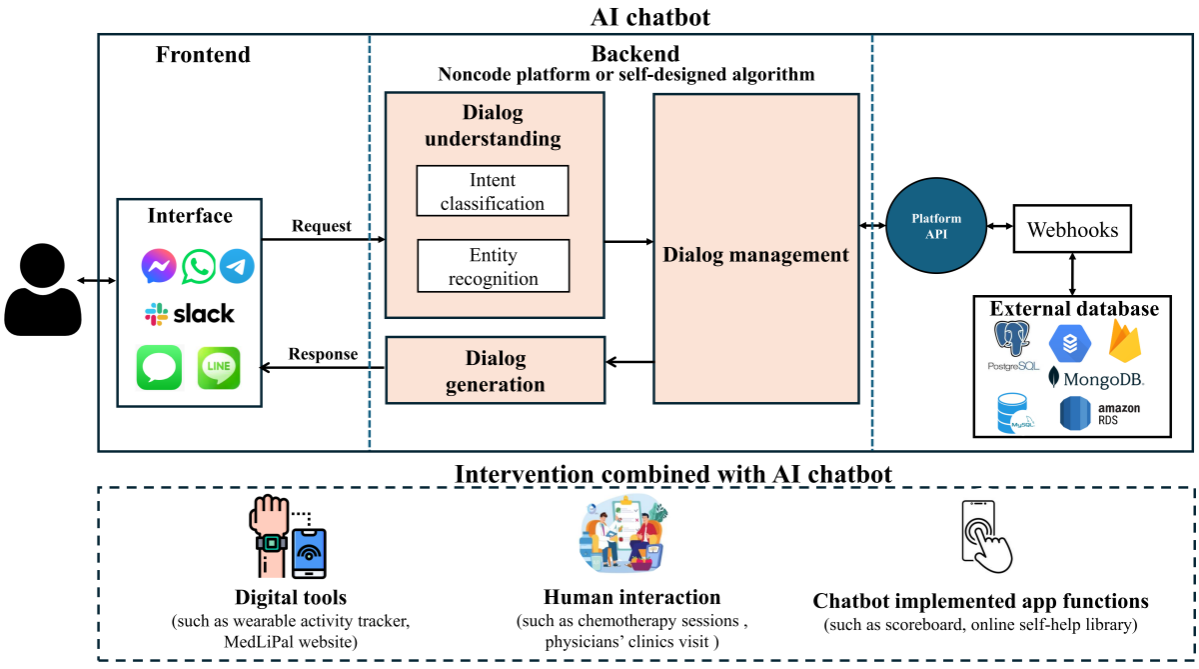
Architecture for the development of AI chatbots. This is a comprehensive picture derived from all the selected studies. Not every study reported the details of each module. AI: artificial intelligence; API: application programming interface.

Table S7 in Multimedia Appendix 2 also provides a summary of the intervention characteristics. Regarding chatbot use, most studies used AI chatbots for 1-time use (10/43, 23.26%), followed by 8 weeks (7/43, 16.28%), 4 weeks (6/43, 13.95%), 12 weeks (5/43, 11.63%), 1 week (5/43, 11.63%), and 2 weeks (3/43, 6.98%). A small number of studies ranged from 3 days [42], 9 days [43], 3 weeks [44], 6 weeks [20], 24 weeks [19,45] to 48 weeks [46]. In terms of intervention modalities, most studies used a text-based chatbot alone (27/43, 62.79%; Table S5 [Study description] in Multimedia Appendix 2). Among them, some studies compared chatbots with other interventions, such as usual care by humans [19] and virtual humans or human teletherapists [24]. Design-focused studies explored personalization differences [47], reward structures [45], and emotional support and self-disclosure [23] to enhance chatbots’ performance. In addition, a subset of studies (16/43, 37.21%) integrated AI chatbots with other components as part of multicomponent interventions, including digital tools, human interaction, and chatbot-implemented app functions (Figure 2 and Table S5 in Multimedia Appendix 2). Digital tools included exergames [37], the MedLiPal website [48], and wearable activity trackers (WATs). In particular, several studies (4/7, 57.14% [20,35,37,38]) technically integrated WATs with AI chatbots, enabling automatic data sharing. In others (3/7, 42.86% [46,48,49]), participants used WATs for self-monitoring to achieve goals set by the chatbots. AI chatbots were also integrated with human-delivered interventions, such as chemotherapy sessions [35], weight management programs in hospitals [41], physicians’ clinic visits [45], the StudentBodies web-based program [39], family-based lifestyle modification programs [40], and remote traditional therapy [50,51]. Furthermore, several studies implemented AI chatbots within stand-alone applications that incorporated additional supportive features. These included a calendar and scoreboard [34], an online self-help library and e-book collection [33], an online smoking cessation diary [45], and self-care practice tools [18].

### Question 1: What Are the Most Commonly Targeted Health Behaviors in Text-Based AI Chatbots?

Most studies focused on one health behavior (OHB; 29/43, 67.44%), and 14 studies addressed multiple health behaviors (MHBs; 14/43, 32.56%). PA was widely explored (18/43 with 8 OHB and 10 MHB), followed by stress management (16/43 with 10 OHB and 6 MHB), diet (11/43 with 1 OHB and 10 MHB), smoking cessation (7/43 with 6 OHB and 1 MHB), sleep (7/43 with 1 OHB and 6 MHB), weight management (6/43 with 2 OHB and 4 MHB), alcohol (1/43 OHB), and sedentary (1/43 MHB; Table 1).

**Table 1:** Targeted health behaviors (N=43).

Target behavior		References	Values, n (%)
**Single behaviors (n=29, 67.44%)**			
	Stress management	[23,24,42,50-56]	10 (23.26)
	Physical activity (PA)	[16,20,37,38,44,47,57,58]	8 (18.60)
	Smoking cessation	[19,34,43,45,59,60]	6 (13.95)
	Weight management	[17,41]	2 (4.65)
	Diet	[36]	1 (2.33)
	Sleep	[61]	1 (2.33)
	Alcohol	[62]	1 (2.33)
**Multiple behaviors (n=14, 32.56%)**			
	Diet and PA	[35,48,49,63]	4 (9.30)
	Diet and weight management	[39,64]	2 (4.65)
	Diet, PA, sleep, and stress management	[65,66]	2 (4.65)
	Diet, PA, sleep, and weight management	[40]	1 (2.33)
	Diet, PA, sleep, stress management, and sedentary	[67]	1 (2.33)
	Sleep and stress management	[18,33]	2 (4.65)
	PA and stress management	[68]	1 (2.33)
	PA, smoking cessation, and weight management	[46]	1 (2.33)

### Question 2: What Roles, Theoretical Foundations, and Behavior Change Techniques Are Applied in Text-Based AI Chatbots, Supporting Health Behavior Change Interventions?

#### Overview

We classified the AI chatbot for health behavior change into 2 roles and summarized theoretical foundations as well as corresponding functionalities (Table 2).

**Table 2:** Artificial intelligence chatbot roles, theoretical foundations, and health behavioral change techniques^a^.

Paper		Theoretical foundation	A	B	C	D	E	F	G	H	I	J	K	L	M	N	O	P
**Routine coach and on-demand assistant (n=4)**																		
	Davis et al (2020) [49]	NR^b^	✓^c^	✓	✓	✓			✓									
	Hassoon et al (2021) [38]	NR	✓	✓								✓						
	Maher et al (2020) [48]	NR	✓	✓	✓	✓			✓									
	Perski et al (2019) [34]	Mohr’s model of supportive accountability					✓		✓									
	Category sum	—^d^	3	3	2	2	1	0	3	0	0	1	0	0	0	0	0	0
**Routine coach (n=27)**																		
	Albino de Queiroz et al (2023) [35]	CBT^e^		✓					✓									
	Albers et al (2023) [47]	NR	✓				✓	✓										✓
	Almusharraf et al (2020) [59]	MI^f^	✓			✓					✓							
	Brown et al (2023) [60]	MI	✓		✓				✓		✓							
	Cameron et al (2018) [33]	NR		✓	✓	✓												
	Catellani et al (2023) [37]	Multiple theory 1^g^					✓											
	Daley et al (2020) [52]	CBT and positive psychology		✓	✓	✓												
	Dhinagaran et al (2021) [67]	COM-B^h^			✓	✓												
	Figueroa et al (2021) [16]	Multiple theory 2^i^	✓															
	Fitzsimmons‐Craft et al (2022) [39]	CBT		✓	✓	✓				✓					✓			
	Medeiros et al (2022) [42]	Emotion self-regulation			✓	✓											✓	
	Legaspi et al (2022) [18]	NR	✓	✓		✓			✓						✓			
	Karhiy et al (2023) [24]	NR				✓												
	Meng and Dai (2021) [23]	NR		✓	✓													
	Moore et al (2024) [57]	COM-B and the Theoretical Domains Framework	✓	✓		✓				✓							✓	
	Piao et al (2020) [44]	HFM^j^	✓	✓					✓	✓		✓						
	Piao et al (2020) [58]	HFM	✓	✓					✓	✓		✓						
	Rahmanti et al (2022) [64]	COM-B	✓	✓	✓	✓	✓		✓			✓						
	Sia et al (2021) [66]	NR	✓		✓	✓			✓			✓					✓	
	Sun et al (2023) [68]	MI and graded exercise therapy	✓			✓												
	Albers et al (2023) [43]	NR	✓	✓	✓	✓		✓	✓		✓	✓						
	Aarts et al (2022) [61]	NR		✓														
	Holmes et al (2019) [17]	NR		✓	✓													
	Griol et al (2022) [63]	NR		✓	✓	✓	✓											
	De Nieva et al (2020) [53]	CBT	✓	✓		✓				✓								
	Carrasco-Hernandez et al (2020) [46]	CBT and MI		✓	✓		✓										✓	
	Masaki et al (2019) [45]	NR	✓	✓	✓	✓												
	Category sum	—	14	17	14	16	5	2	8	5	3	5	0	0	2	0	4	1
**On-demand assistant (n=12)**																		
	Stephens et al (2019) [41]	Multiple theory 3^k^	✓	✓	✓													
	Danieli et al (2021) [50]	CBT		✓	✓	✓												
	Danieli et al (2022) [51]	CBT		✓	✓	✓												
	Fadhil et al (2019) [65]	CBT		✓	✓	✓			✓									
	Larizza et al (2023) [40]	NR	✓	✓	✓	✓												
	Olano-Espinosa et al (2022) [19]	5A clinic practice guideline			✓	✓				✓		✓						
	Alghamdi et al (2021) [36]	Multiple theory 4^l^		✓								✓	✓					
	To et al (2021) [20]	COM-B	✓	✓	✓		✓		✓	✓		✓					✓	
	Durden et al (2023) [54]	CBT	✓	✓		✓				✓								
	Forman-Hoffman et al (2023) [55]	CBT	✓	✓	✓	✓				✓								
	Hoffman et al (2023) [56]	CBT	✓	✓	✓	✓				✓								
	Prochaska et al (2021) [62]	CBT	✓	✓	✓	✓				✓								
	Category sum	—	7	11	10	9	1	0	2	6	0	3	1	0	0	0	1	0
	Total number	—	24	31	26	27	7	2	13	11	3	9	1	0	2	0	5	1

^a^A: goals and planning; B: feedback and monitoring; C: social support; D: shaping knowledge; E: natural consequences; F: comparison of behavior; G: associations; H: repetition and substitution; I: comparison of outcomes; J: reward and threat; K: regulation; L: antecedents; M: identity; N: scheduled consequences; O: self-belief; P: covert learning.

^b^NR: not reported.

^c^✓: The study reported the results of this theme or subtheme.

^d^Not applicable.

^e^CBT: cognitive behavioral therapy.

^f^MI: motivational interviewing.

^g^Multiple theory 1: elaboration likelihood model, the self-regulatory model of message framing, the regulatory focus theory, and theories of emotions.

^h^COM-B: capability, opportunity, motivation—behavior.

^i^Multiple theory 2: MI, behavioral activation, acceptance and commitment therapy, and solution-focused brief therapy.

^j^HFM: habit formation model.

^k^Multiple theory 3: CBT, MI, and emotionally focused therapy.

^l^Multiple theory 4: chronic-disease extended model extending from the health belief model, the theory of planned behavior, diffusion of innovation theory, social norms theory, and the transtheoretical model.

#### Roles: Routine Coach and On-Demand Assistant

We classified the chatbot into 2 roles based on the intervention dosage (ie, use frequency and duration per interaction), specifically as a routine coach and an on-demand assistant. AI chatbots mostly played 1 role, such as routine coach (27/43, 62.79%) and on-demand assistant (12/43, 27.91%), whereas 4 studies integrated 2 roles (4/43, 9.30%; Table 2). Specifically, routine coaches delivered support in a defined use frequency and duration per interaction, such as 4 times per week, and each focused on 1 of 4 targeted health behaviors [67]. In contrast, on-demand assistants offer support with flexible frequency and intensity, allowing patients to contact the chatbot anytime and anywhere and determine the duration and frequency of interactions themselves [19].

#### Theoretical Foundation

Most studies incorporated a theoretical foundation to guide chatbot design strategies (28/43, 65.12%), with 20 studies applying a single theory and 8 studies using an integrated theoretical approach (Table 2 and Table S8 in Multimedia Appendix 2 [16-20,23,24,33-68]). Cognitive behavioral therapy (CBT; 13/28, 46.43%), MI (6/28, 21.53%), and the capability, opportunity, motivation—behavior framework (4/28, 14.29%) were primarily used, either individually or in combination with other theories.

CBT is a form of psychotherapy based on the concept that people’s thinking influences their emotions and behaviors [76]. MI is a client-centered, directive therapeutic style to discover the client’s own motivation for making changes by guiding clients to explore and resolve ambivalence [50,77]. Finally, capability, opportunity, motivation—behavior is a framework for understanding and changing behavior that posits capability, opportunity, and motivation.

#### Behavior Change Techniques

Because the functions of AI chatbots varied across studies, we mapped them onto the existing BCT taxonomy (version 1) [72] to enable cross-study comparisons (Table 2). The taxonomy included 16 themes and 93 techniques. We conducted a deductive coding process to code each chatbot function based on these 93 techniques and labeled a cluster if at least 1 technique within it was used. For example, if a chatbot provided automated, tailored feedback on reports and behavioral activity [35], we coded this as the technique “2.7 Feedback on outcome(s) of behavior,” which falls under the “Feedback and Monitoring (B)” cluster. Accordingly, we indicated in the table that the study used at least 1 technique within that cluster. The coding was conducted by 1 primary extractor (LF) and validated by 2 additional reviewers (Conrad Ma and Shreya Sanghvi). Any discrepancies were resolved through discussion among all 3 reviewers. The interrater reliability was 81.70%.

Across all included studies, the most frequently applied clusters were goals and planning (A, 24/43, 55.81%), feedback and monitoring (B, 31/43, 72.09%), social support (C, 26/43, 60.47%), and shape knowledge (D, 27/43, 62.79%). No study used the techniques of antecedent (L) and scheduled consequences (N). This pattern shows that AI chatbots currently prioritize conversational, scalable, and digitally applicable techniques, while environmental restructuring and reinforcement-based strategies remain underused due to resource demands and limited environmental control.

#### BCTs by Roles

Goals and planning, social support, and shaping knowledge were commonly used in both routine coach and on-demand assistant (Table 2). The AI chatbot supported users in setting behavior goals and creating action plans [16,44,47,58], as well as reviewing behavior goals using historical data [20]. Additionally, AI chatbots provided social support (emotional or unspecified) in various ways, including personalized motivational dialogue [46], free-form responses [60], nurse contact information [33], and expressive elements such as sending emojis, icons, GIFs, gamification [52], and images [67]. Other strategies included providing a crisis hotline [36] and a 24-hour on-call [19]. Finally, shaping knowledge primarily involved providing clear instructions on how to perform the behavior [18,24,33,38,42,57,66,68]. For example, chatbots prompted users to share stressful experiences and then offered personalized suggestions, such as planning their day or reframing their mindset [18,42]. The main difference between routine coaches and on-demand assistants was the use of feedback and monitoring techniques (B), applied by 91.67% (11/12) of on-demand assistants and 62.96% (17/27) of routine coaches. This may be explained by the characteristics of on-demand assistants, which allow for continuous interaction, real-time behavior tracking, immediate feedback delivery, and progress reinforcement at any time. Routine coaches typically delivered feedback based on 1-time interactions, such as mood reflection [52], behavioral reporting [17,63], sleep diary [61], and gratitude journaling [53]. In contrast, on-demand assistants provided personalized feedback through active monitoring of health behavior level [20,40,65], mood status [41,55,56,62], and overall health behavior change progress [19,35,36].

### Question 3: What Technologies Are Used to Develop Text-Based AI Chatbots for Health Behavior Change?

#### Workflow of AI Chatbots

Figure 2 summarizes the workflow of AI chatbots, including the frontend module, backend module, and external service module. The process generally involves the following steps: (1) the user sends messages through the frontend interface (eg, social messaging platform, web-based interface, or SMS); (2) the frontend interface forwards the message to the backend (eg, noncode platforms and self-designed algorithms) hosted on the research team’s server; and (3) the backend system processes the messages, including dialogue understanding, management, and generation. The function of dialogue understanding is to extract meaning from the user input, such as intents and entities. Dialogue management involves domain-specific knowledge for tailoring replies. The text generation provides output to the user [78]; (4) the generated replies are sent and delivered back to the user through the frontend interface. If the selected intent requires additional operations, such as retrieving data from the external database, the platform sends a request to the webhook through its application programming interface (API). The webhook then processes the intents and returns a response to the platform, which subsequently delivers it to the user in an understandable format. Researchers can create custom webhooks to handle more complex intents.

Based on the development workflow, we classified AI chatbot technologies into 3 types: noncode platforms (23/43, 53.49%), self-designed algorithms (8/43, 18.60%), and stand-alone apps (12/43, 27.91%). These approaches can inform and guide researchers in developing AI chatbots for future use (Table S9 in Multimedia Appendix 2 [16-20,23,24,33-68]).

#### Noncode Platform

Noncode platforms offer built-in AI algorithms, allowing researchers to develop chatbots without writing code. Among the 23 studies using noncode platforms, the most commonly used platforms were Google Dialogflow (8/23, 34.78%) and IBM Watson (8/23, 34.78%), followed by X2AI (2/23, 8.70%) and Chatfuel (2/23, 8.70%). These AI chatbots were primarily accessed through social messaging platforms, such as Facebook Messenger [23,35,42,67], Slack [48,49], and SMS [16,39,41]. Several studies connected the chatbot to external systems to customize historical data through APIs. Examples include Google Cloud Functions and databases [35], PostgreSQL [40], and MongoDB [16,17,42,63].

#### Self-Designed Algorithms

Self-designed algorithms require researchers to develop their own AI models, allowing for greater customization of chatbot capabilities. Among 8 studies, some applied Bayes’ theorem to assess individual needs and used natural language processing to enhance the chatbot’s understanding and response capabilities [19]. Other approaches included combining GPT-2 XL with natural language processing for dialogue understanding and generation [60], deep reinforcement learning [37,43], supervised goal-based models [50,51], self-learning algorithms [46], and the Microsoft Bot Framework [33]. These custom backend systems were integrated with frontend interfaces through APIs, including Telegram [19], web-based interfaces, and SMS text messaging [38], as well as connected with external databases, such as MySQL database [33].

#### Stand-Alone Applications

Stand-alone apps encapsulate the entire workflow of AI chatbots, as illustrated in Figure 2. In total, 12 studies used stand-alone apps, such as Woebot [53-56,62], Wysa [18], Vitalk [52], Smoke-Free [34], and PsyMe [37]. Each app offered unique features tailored to specific functions. For example, Woebot emphasized helping users develop emotional regulation skills and support mood monitoring and management through conversations. Wysa was not only an AI chatbot but also a comprehensive mental health app, offering additional features such as access to a human talk therapist, stress management techniques, a journal for gratitude, and an international distress signal feature to seek help.

### Question 4: How to Validate Text-Based AI Chatbot Performance in Health Behavior Change?

#### Performance Validation Framework

We mapped the measurements from all included studies onto the digital health scorecard framework [73] and engagement framework [74] to identify existing evidence and current gaps in validating the performance of AI chatbots in health behavior change. The digital health scorecard framework included 4 domains: technical, clinical, usability, and cost. Technical refers to testing if the AI chatbots actually perform to their self-proclaimed functionality with accuracy and precision. Clinical was operationalized as the critical appraisal of evidence demonstrating whether the AI chatbots impact the defined health behavior change outcomes. Usability refers to how easily an AI chatbot can be used for its intended purpose and the minimal effort required to complete tasks. Cost refers to the price for user access, technology lifecycle expenses, and integration costs within clinical workflows. However, these domains primarily assess how well a chatbot performs in influencing changes in health behaviors. Therefore, we incorporated engagement into the framework to capture user engagement between human-AI interaction, reflecting motivational and relational aspects that the other domains do not address. Engagement with digital behavior change interventions includes (1) the extent of use, such as amount, frequency, duration, and depth; and (2) a subjective experience characterized by attention, interest, and affect [74].

**Figure 3:**
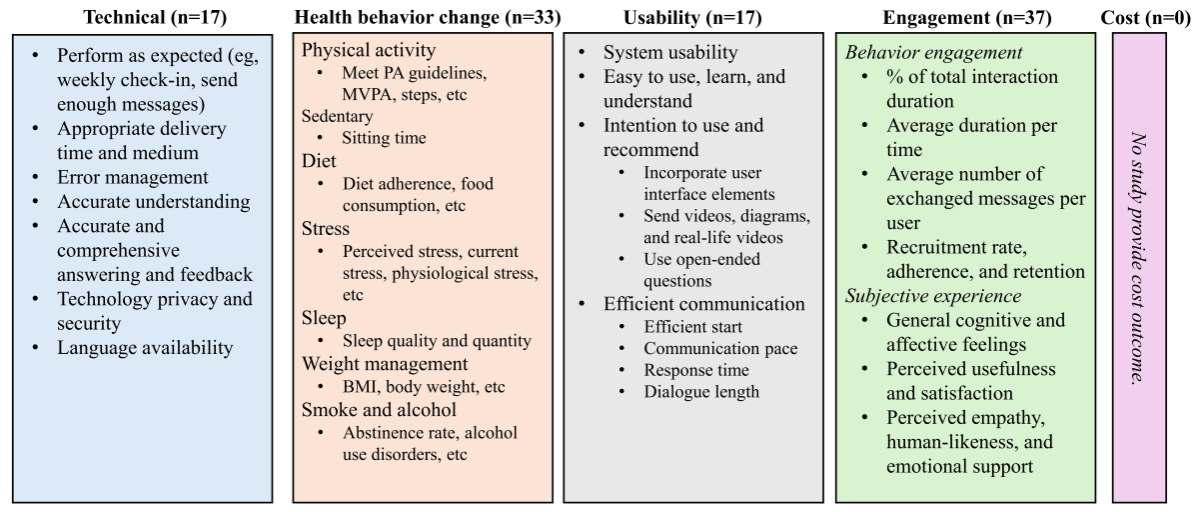
Artificial intelligence chatbots on the health behavior change validation framework. MVPA: moderate-to-vigorous physical activity; PA: physical activity.

In terms of the coding process, first, we conducted deductive coding to map the measures from each study onto 5 domains: technical performance, health behavior change, usability, cost, and engagement. For example, if a study measured “technical feedback from users, ease of use, ease of learning, perceived usefulness, and satisfaction,” we mapped “technical feedback from users” to the technical domain; “ease of use” and “ease of learning” to the usability domain; and “perceived usefulness” and “satisfaction” to the engagement domain, based on the definitions of each domain [65]. This process was conducted by 1 primary extractor (LF) and independently validated by 2 additional reviewers (Conrad Ma and Shreya Sanghvi). Any discrepancies were resolved through discussion among all 3 reviewers. The interrater reliability was 90.18%. In the next step, within each domain, we conducted inductive coding to group similar measurements and identify representative metrics, as shown in Figure 3.

Across 43 studies, 17 assessed technical performance, 33 evaluated health behavior change outcomes, 17 examined usability outcomes, and 37 measured engagement outcomes. None of the studies reported cost-related evidence (Figure 3).

#### Question 4.1: What Measures Are Used to Assess Technical Performance, Usability, and Engagement?

##### Technical

In Figure 3, the technical performance of AI chatbots for health behavior change was evaluated across several aspects. The first metric was performance as intended, such as supporting weekly check-ins [49] and delivering a sufficient number of messages [20]. Delivery time and medium were evaluated to determine whether chatbots provided information promptly and through appropriate channels [67]. Error management focused on how effectively chatbots handled unexpected issues [17,20,33]. Several studies also assessed the chatbot’s ability to accurately understand user input [33,46,49,53,63,65,66], as well as the provision of accurate and comprehensive information and feedback [40,63,68]. Language availability emerged as a key metric influencing chatbot performance, including the need for additional language options [63] and maintaining language consistency to generate appropriate, user-aligned responses [61,66]. Studies also highlighted that the use of local languages enhanced human connection and personalization [65], while simple and clear language improved user interaction and accessibility [67]. Finally, privacy and security concerns related to the technology were also important metrics to consider when adopting AI chatbots for user interventions [16].

##### Usability

In terms of usability, 4 studies measured general usability using the System Usability Scale, which includes items such as “I thought the system was easy to use.” In total, 3 of those reported above-average industry scores (>68), including 88.2 [33], 84.8 [17], and 79.6 [35], while 1 reported a below-average score of 61.6 [20]. Beyond the System Usability Scale, some studies included items measuring ease of use, ease of learning, and ease of understanding [16,17,35,44,64,65,67,68], as well as intention to use [16,57,58,67,68] and recommendations to others [62]. Usability was also assessed through efficient communication, including smooth onboarding [17,33], suitable interaction pace and response time [17,33,57,64,67], and appropriate conversation length to maintain user engagement [61]. Researchers also identified several features that enhance usability, including human touch and user interface elements [65]; using multimedia such as videos, diagrams, and real-life examples; posing open-ended questions [57]; and allowing free-text input for communication [43].

##### Engagement

In Figure 3, behavioral engagement refers to engagement intensity and longevity. Most studies reported moderate interaction duration, including 50% [55], 62.5% [35], 66.78% [45] of the total intervention period. The average interaction duration per time was typically less than 30 minutes, such as 5.1 (SD 7.4) minutes [41], 12.5 (SD 15.62) minutes [59], and 21.3 (SD 14.0) minutes [20]. The average number of exchanged messages per user throughout the entire interventions varied between 245.1 [52] and 547.3 [59]. In addition, engagement at different stages was measured through recruitment (how many new individuals are added to a project within a specific time frame), adherence (the extent to which a person’s behavior corresponds with agreed-upon recommendations from a care provider [79,80]), and retention (the extent to which the participants completed the study). The recruitment rate ranged from a high of 82% among inactive community-dwelling adults aged 45-75 years [48] to lower rates of 60% among young adults with eating disorders aged 18-30 years [39] and 55.1% among healthy adults aged 21 years and older [67]. Additionally, low adherence (<70% [48]) was reported in 2 studies, with participants completing an average of 63% (6.9/11) of weekly check-ins in one study [48] and 61% (6.7/11) in another [49]. A slight decline in weekly adherence was also observed, decreasing from 77% in week 1 to 69% in week 4 [62]. In contrast, higher retention rates were reported in the other 2 studies (>70% [48]), including 90% [48] and 93% [67].

In terms of subjective experience, several trials reported positive attitudes and acceptance [43,47,53,57], as well as feelings of low frustration, enjoyment [59], interesting [37], attractiveness, stimulation, novelty [17,57], and openness [18]. In addition, other metrics, such as feelings of helpfulness [39,41,51,59] and satisfaction [16,51,65,67], were also commonly reported. Furthermore, several studies highlighted negative perceptions of relational quality, such as complications [16], lack of empathy [18,60], limited human likeness [16,18,66], low affective support [53,57,66], robotic or unfriendly [17], lack of authenticity [47], and low motivational, as well as low emotional support [23].

#### Question 4.2: What Are the Health Behavior Change Outcomes?

##### Behavior Change Outcome Overview

Figure 3 illustrates the primary health behavior change outcomes, and Table S10 in Multimedia Appendix 2 [18-20,23,24,34-39,41-43,45-58,60,62,66-68] provides exploratory findings on the efficacy of AI chatbots for each outcome. In total, 33 of the included studies reported health behavior–related outcomes, yielding a total of 120 comparisons. To quantify the magnitude of change across interventions or pre- and postassessments, effect sizes were expressed as either Hedges *g*, odds ratios (ORs), or Cohen *d*. Hedges *g* was calculated when means and SDs were available, whereas ORs were used when only categorical data were reported. Cohen *d* from the original study was used when insufficient information was available to calculate Hedges *g*. Studies reporting Cohen *d* are indicated in Table S10 in Multimedia Appendix 2. According to Cohen conventions, a medium effect of 0.5 is visible to the naked eye of a careful observer [81].

##### Positive Changes

Among 33 studies with 120 comparisons, 81.67% (98/120) reported positive changes in promoting health behaviors. Positive changes refer to either statistically significant or nonsignificant improvements. However, only 35.83% (43/120) of these comparisons demonstrated observable positive changes with a moderate or larger effect size (Hedges *g* or OR or Cohen *d*>0.5). Moreover, it should be noted that most positive findings were observed in PA, smoking, stress management, and diet, indicating the need for more evidence in weight management, sleep, alcohol use, and sedentary behavior. Additionally, only a small portion of studies were RCTs (14/33, 42.42%), and the populations were primarily nonclinical adults (21/33, 63.64%).

##### Effectiveness in Real-World Settings

Only 4 of 33 (12.12%) studies evaluated the effectiveness of AI chatbots in real-world settings, all of which were non-RCTs focusing on stress reduction. Among them, the strongest clinically significant decrease in stress was *g*=–0.90 (95% CI –0.97 to –0.83) [52]. When comparing effectiveness by location, use patterns, and emotional status, studies found no significant differences between medically underserved areas and nonmedically underserved areas (t_253_=0.30; *P*=.77; *d*=0.04, 95% CI –0.23 to 0.30) or between mental health provider shortage areas and nonmental health provider shortage areas (t_253_=–1.39; *P*=.17; *d*=–0.18, 95% CI –0.44 to 0.07) [55]. Efficient users, those with lower behavioral engagement but stronger therapeutic alliance, achieved greater stress reductions (*g*=–0.60, 95% CI –0.86 to –0.33) than typical users (*g*=–0.25, 95% CI –0.47 to –0.03) and early users (*g*=–0.44, 95% CI –0.71 to –0.17) [56]. Participants with elevated mood symptoms at baseline experienced the greatest stress reduction (*g*=–0.68, 95% CI –0.93 to –0.44) compared with those with low mood symptoms (*g*=–0.28, 95% CI –0.53 to –0.02) [54].

##### Long-Term Efficacy

A total of 5 (5/33, 15.15%) studies evaluated follow-up efficacy after the intervention, including smoking cessation [45,60], stress [50,51], and diet-related outcomes [39]. Continuous smoking-related improvements were observed. One study reported confidence (*g*=0.56, 95% CI 0.27-0.84), importance (*g*=0.24, 95% CI –0.03 to 0.52), and readiness to quit smoking (*g*=0.17, 95% CI –0.10 to 0.45) from baseline to 1-week follow-up compared to a single postsession measurement [60]. There was also strong and sustained smoking cessation across multiple follow-up points, with large effects at 12 weeks (*g*=1.40, 95% CI 1.03-1.78), 24 weeks (*g*=1.74, 95% CI 1.31-2.17), and 52 weeks (*g*=1.24, 95% CI 0.88-1.61) compared to 9 weeks after the intervention [45]. However, 2 RCTs did not find a consistent reduction in stress up to 12 weeks after an 8-week intervention in either the chatbot-only group [51] or the multicomponent intervention integrated with the chatbot group [50,51]. Similarly, for nonclinical eating disorder symptoms, the effect size between the intervention and control groups declined over time after the 4-week intervention (12-week: *g*=–0.41, 95% CI –0.63 to –0.20; 24-week: *g*=–0.20, 95% CI –0.41 to 0.01) [39].

##### Intervention Duration

Intervention duration appears to be an important factor influencing the efficacy of AI chatbots on health behavior change (4/33, 12.12%). A pre- and poststudy found that longer intervention duration (>6 weeks) yielded small but additional benefits across multiple behaviors among middle-aged and older adults, including weight loss (6 weeks: *g*=–0.06; 12 weeks: *g*=–0.07), waist circumference (6 weeks: *g*=–0.06; 12 weeks: *g*=–0.13), diet adherence (6 weeks: *g*=2.04; 12 weeks: *g*=2.06), and PA (6 weeks: *g*=0.32; 12 weeks: *g*=0.39) [48]. Similarly, another RCT reported that the percentage of participants increasing their metabolic equivalent of task scores rose from mid-intervention (*g*=–0.14, 95% CI –1.35 to 1.07) to the end of the 48-week intervention (*g*=0.06, 95% CI –0.87 to 0.98) [46]. Differences in smoking cessation outcomes related to intervention duration were also observed across 2 RCTs. A longer intervention duration of 48 weeks was associated with higher odds of biochemically validated abstinence in the chatbot group compared with the control group (OR 1.01, 95% CI 0.18-1.84) [46], whereas a shorter 24-week duration showed lower odds (OR 0.84, 95% CI 0.31-1.37) [19].

##### Intervention Modalities

In total, 9 of 33 (27.27%) studies have examined the impact of chatbot modalities on health behavior change outcomes, with all of them being RCTs. First, text-based chatbots performed worse than other modalities, such as video-based chatbots, virtual humans, and human coaches. A text-based AI chatbot showed a smaller increase in PA (*g*=0.35, 95% CI –0.39 to 1.10) compared with a video-based chatbot (*g*=1.14, 95% CI 0.34-1.94) after a 4-week intervention [38]. Similarly, the text-based chatbot demonstrated the smallest effect size in stress reduction (Cohen *d*=0.36) compared with the virtual human (Cohen *d*=0.52) and teletherapy (Cohen *d*=0.54) groups [24]. A text-based chatbot-only intervention (*g*=–0.34, 95% CI –1.33 to 0.65) also performed worse in reducing stress compared with traditional therapy (*g*=–0.71, 95% CI –1.44 to 0.03) [51]. Furthermore, participants who believed that they were interacting with a bot experienced a smaller reduction in stress than those who knew they were interacting with a human [42]. In addition, multicomponent interventions that combined text-based chatbots performed better than traditional therapy or other digital tools alone. For example, traditional therapy plus an AI chatbot led to greater stress reduction after an 8-week intervention compared with traditional therapy alone [50,51]. Similarly, combining psychopharmacological therapy with a digital therapeutic solution including an AI chatbot produced better stress reduction than psychopharmacological therapy alone (*g*=0.13, 95% CI –0.28 to 0.53) [46]. Finally, regarding chatbot design features, chatbots incorporating cues and intrinsic or extrinsic rewards significantly increased PA compared with a control chatbot without intrinsic rewards (t_104_=2.12; *P*=.04) [58]. Personalized examples were linked to a significant increase in motivation (*g*=0.98, 95% CI 0.60-1.36) but a significant decrease in self-efficacy for PA engagement (*g*=–2.57, 95% CI –3.22 to –1.92) [47].

##### Engagement

Engagement, encompassing both behavioral engagement and subjective experience, emerged as a significant factor in promoting health behavior change (6/33, 18.18%). Most studies found that strong engagement was associated with positive outcomes, with the exception of one study [52]. High engagers (≥8 weekly check-ins) demonstrated greater increases in PA (high: *g*=0.65, 95% CI –0.14 to 1.44; low: *g*=0.51, 95% CI –0.15 to 1.18) but lower improvements in diet adherence (high: *g*=2.60, 95% CI 1.55-3.64; low: *g*=3.66, 95% CI 2.54-4.78) compared with low engagers [49]. Efficient engagers, those with lower behavioral engagement but stronger therapeutic alliance, had significantly greater reductions in stress than other user groups (*g*=–0.60, 95% CI –0.86 to –0.33) [56]. Intensive users (>4 contacts and >30 minutes of total interaction time) achieved higher quit rates than nonintensive users in both the chatbot intervention group (*g*=1.12, 95% CI 0.55-1.68) and usual care group (*g*=0.52, 95% CI 0.02-1.02) [19]. In addition, a causal mediation analysis explained that higher message involvement positively influenced PA intention through increased feelings of calmness (β=.07; *P*=.003) and greater hope (β=.44; *P*<.001) [37]. Finally, subjective feelings, such as the emotional support provided by AI chatbots, significantly reduced perceived stress through perceived supportiveness, underscoring the importance of subjective engagement experiences [23].

##### Sleep, Alcohol, Sedentary, and Weight Management

There were a small number of studies examining the efficacy of AI chatbots on sleep (n=2), weight management (n=4), alcohol use (n=1), and sedentary behavior (n=1). All of these were non-RCTs, except for the study by Carrasco-Hernandez et al [46]. First, there was no consistent evidence that AI chatbots effectively improved sleep quality or sleep quantity. One study found no significant effects on sleep quality (*g*=0.02, 95% CI –0.34 to 0.38) or sleep duration, with the proportion of short sleepers increasing by 6% after a 4-week intervention [67]. In contrast, another study reported a modest 3% improvement in sleep quality after a 1-week intervention [66]. Additionally, weight management appeared to be more challenging to change through chatbot interventions. A pre- and poststudy observed only small effects on weight loss (*g*=–0.07, 95% CI –0.57 to 0.43) and waist circumference reduction (*g*=–0.13, 95% CI –0.63 to 0.37) after 12 weeks among middle-aged and older adults [48]. Similarly, other studies found no significant changes in BMI at 6-week postintervention (*g*=–0.01, 95% CI –0.27 to 0.24) [20], 24-week mid-intervention (*g*=–0.05, 95% CI –0.45 to 0.35) [46], and 48-week postintervention (*g*=0.13, 95% CI –0.28 to 0.53) [46]. Furthermore, the findings for alcohol use and sedentary behavior were relatively positive, showing a significant reduction in alcohol use disorder symptoms (*g*=–0.42, 95% CI –0.81 to –0.03) [62] and a 32 minutes per day decrease in sitting time [67].

## Discussion

### Principal Findings

The rapid advancement of AI and increased computational power have significantly expanded the potential applications and advantages of AI chatbots in facilitating health behavior change. This study aimed to provide an up-to-date overview of AI chatbot applications in this domain, along with practical guidance for their development and implementation. Consistent with prior research reviews [10,25,82], PA has emerged as a prominent focus area. This might be due to the need for a scalable intervention to solve the pandemic PA problems [83]. Health behavior change chatbots were classified as routine coaches (predefined frequency and intensity) and on-demand assistants (no specific frequency and intensity). Routine coaching offers a low-cost alternative that can supplement human therapists by providing guidance during their unavailable periods. On-demand assistants allow users to self-monitor and provide timely feedback. The 2 roles address key limitations of conventional interventions by providing more timely, low-cost, and personalized support while also reducing the resource burden on the traditional health care system. Considering theoretical foundations, most AI chatbots have been developed based on CBT and use BCTs such as goal setting and planning, feedback and monitoring, social support, and shaping knowledge. Notably, compared with routine couches, on-demand support chatbots rely more heavily on CBT as well as feedback and monitoring techniques. To achieve these functions, 3 main approaches have been used to develop AI chatbots: noncode platforms, self-designed algorithms, and stand-alone applications. Most studies used noncode platforms, such as Google Dialogflow and IBM Watson, which were then integrated into popular social messaging interfaces, including Facebook Messenger. These noncode platforms are particularly feasible for health behavior researchers who might lack programming expertise. Thus, it significantly improved accessibility and promoted wider adoption of chatbot interventions in health behavior change (across 262 health care centers [19] and up to 57,214 participants [34]).

We refined the validated digital framework [73] by adding engagement elements [74]. The updated framework, which includes technical, health behavior change, usability, engagement, and cost, captures all major measures of assessing the performance of AI chatbots in supporting health behavior change. The findings revealed a notable gap in cost-related evidence and highlighted the need for standardized approaches to calculate a global performance score. Such a standardized benchmark would help distinguish between high- and low-performing AI chatbots and enable cross-study comparisons. Moreover, the exploratory efficacy findings indicated that, although existing studies generally show positive effects of AI chatbots on health behavior change, evidence supporting clinically observable outcomes remains limited. Additionally, most studies have been conducted with nonclinical adult populations (aged 19-64 years), using nonrandomized or short-term trials (≤4 weeks), and have primarily focused on PA, stress management, smoking, and diet. Therefore, researchers should be cautious when applying these findings to clinical settings.

There is also a lack of evidence on the effectiveness of AI chatbots in real-world settings and their long-term efficacy in supporting health behavior change. With regard to intervention design and efficacy, a recent meta-analysis found no significant differences in chatbot effectiveness for increasing moderate-to-vigorous PA, daily steps, or fruit and vegetable consumption by intervention duration or intervention components [12]. In contrast, our exploration scoping review identified consistent findings that the longer intervention duration provides additional benefits across multiple behaviors, such as PA, diet, stress management, and weight management. Multicomponent interventions appeared more effective for stress management and food intake than chatbot-only interventions, though findings for PA were inconsistent. Regarding chatbot modalities, a previous meta-analysis reported that text-based chatbots were more efficacious than audio-based chatbots for fruit and vegetable consumption [12]. In contrast, our exploratory scoping review consistently found that text-based chatbots did not outperform other modalities, such as audio-based chatbots, human therapy, and virtual humans, in terms of PA and perceived stress management. This confirmed the statements that AI chatbots are not intended to replace health care professionals or provide treatment, but rather to complement existing care [52]. The inconsistent findings between this scoping review and the previous meta-analysis [12] underscore the need for additional systematic reviews and meta-analyses to provide more up-to-date and definitive conclusions. The exploratory findings also showed that higher engagement with AI chatbots was associated with greater improvements in health behavior outcomes, including increased PA, better diet adherence, lower perceived stress, and higher quit rates. These findings support previous research, indicating that engagement is a key factor in promoting health behavior change [83]. Finally, the minimal effects on weight management outcomes found in this scoping review were consistent with findings from the broader digital health intervention literature [84,85]. This likely reflects the physiological constraints of weight loss [46] and the fact that most chatbot interventions have targeted activity-related outcomes rather than weight outcomes. Despite these insightful findings, researchers should interpret this conclusion with caution, as it is exploratory and drawn from a broad scoping review rather than a rigorous systematic review and meta-analysis. Moreover, the evidence is based on small and fragmented samples across diverse health behaviors, which limits the strength of conclusions for any single behavior. Future systematic reviews and meta-analyses covering a wider range of health behaviors are needed to provide stronger and more definitive evidence.

### Implications

#### Practical Implications

AI chatbot shows benefits in promoting health behaviors among nonclinical adult populations, including PA, smoking, stress management, and diet. The chatbot can be strategically leveraged to facilitate health behavior change either as a stand-alone tool or by integrating it into existing programs, serving 2 primary roles: routine coaching and on-demand assistance. Establishing a clear distinction between these roles is critical for determining the appropriate frequency, intensity, and structure of user use. Moreover, researchers can design AI chatbot functionalities based on the synthesized evidence from health behavior change theories and BCTs identified in this scoping review. However, the most effective functionalities remain to be fully explored, and the underlying mechanisms are not yet well understood. Additionally, an accessible approach for health behavior scientists is to use no-code platforms (eg, IBM Watson and Google Dialogflow) or consumer-facing applications (eg, Woebot and Wysa) to develop and deploy AI chatbots for health behavior change interventions. Engagement is a critical factor that requires careful consideration, given the well-documented challenges of sustaining long-term engagement in AI chatbot interventions [34,39]. To address this issue, researchers should develop strategic approaches to maintain user engagement throughout the intervention period. Such strategies may include ensuring high response quality, optimizing interaction length [39], and incorporating visual elements, such as icons and graphs, to enhance user experience and promote sustained participation. It should also be noted that designing such chatbots requires careful consideration of participant characteristics (eg, age, gender, and clinical vs nonclinical populations) and contextual factors (eg, socioeconomic status, digital literacy, cultural norms, and technological environments) to ensure relevance and accessibility, thereby enhancing long-term engagement and achieving targeted outcomes. Finally, future research should incorporate a comprehensive set of evaluation measures encompassing 5 key domains, including technical performance, usability, health behavior changes outcomes, user engagement, and cost, to enable a more rigorous and holistic validation of AI chatbot efficacy.

#### Research Implications

This review summarized only the BCTs and theoretical foundations, underscoring the need for future research to identify the most influential BCTs and to examine how specific techniques (eg, rewards and graphical feedback) influence health behavior change outcomes within particular theoretical frameworks. In terms of technologies, most studies rely heavily on noncode platforms and conventional AI models. This approach might result in limited natural language communication capabilities and several well-documented issues, including insufficient human-like interaction, a lack of affect, empathy, and emotional support. To address these challenges, future research should consider integrating more advanced AI algorithms, such as generative models (eg, GPTs). Examining whether variations in these technologies influence the overall performance of AI chatbots is also important [86]. Additionally, most of the studies included in this review were conducted among Western, educated, industrialized, rich, and democratic populations [87] and nonclinical healthy adults. This limits the generalizability of the findings and practical guidance, as AI chatbot performance may be moderated by factors such as age differences [18,61], digital literacy, app familiarity, linguistic and cultural differences [16], as well as underserved settings [55]. Therefore, future research should focus on designing AI chatbots tailored to diverse demographic groups (eg, clinical populations, youths, and older adults) and contextual factors (eg, digital health equity) to achieve better outcomes across a broader range of populations.

Regarding AI chatbot validation outcomes, more evidence is needed on cost, weight management, sleep, sedentary behavior, and alcohol use. Additionally, more RCTs involving diverse populations, including younger and older adults, clinical populations, and individuals from varied social, economic, and cultural backgrounds, are needed to provide stronger and more comprehensive evidence. There is also a need to establish a gold standard to standardize scoring across different framework domains, including technical, usability, health behavior change, engagement, and cost. For example, a benchmark can be used to determine that when ≥75% of people think the chatbot is useful, it can be regarded as high accuracy (10/10). This enables the aggregation of individual domain assessments into a Global Digital Health Score, which can help validate the quality of AI chatbots and identify effective digital solutions. It can also highlight areas for improvement and inform stakeholders about potential gaps prior to product deployment. In addition to AI chatbot intervention outcomes, implementation outcomes such as reach, adoption, cost-effectiveness, fidelity, maintenance, scalability, and effectiveness also need to be explored. This would enhance the practical relevance of AI chatbots for digital health practitioners, supporting their implementation in real-world settings and improving scalability. Furthermore, more systematic reviews and meta-analyses need to explore the influence of intervention duration, multicomponent designs, and dose-response factors (eg, duration, frequency, and intensity) on AI chatbot performance, particularly given the variations across different health behaviors. Finally, the associations among different measures within the 5 clusters, including technical, usability, health behavior change, engagement, and cost, require further investigation. For example, usability, measured by willingness to continue, was associated with motivation to engage in activities and smoking quitter self-identity [43]. This can help optimize chatbots to better align with user needs, ultimately leading to improved health behavior change outcomes.

### Strengths and Limitations

#### Strengths

This scoping review contributed to previous research in 5 key ways [10,12,25]. First, unlike prior reviews that focused on narrow behavioral domains, this study encompassed a comprehensive range of health behaviors, including PA, diet, sleep, weight management, sedentary behavior, stress management, smoking, and alcohol consumption. We also used an extensive search strategy incorporating synonyms and part-of-speech variations. This approach yielded a substantially larger pool of eligible papers, providing a more holistic understanding of AI chatbots’ role in health behavior change. Second, this review presents a detailed technology workflow for developing AI chatbots, which spans from frontend interfaces, backend architecture, and integration with external systems. By presenting this framework, we offered health behavior researchers, particularly those without computer science expertise, clear guidance on the technical foundations of chatbot implementation. Third, we classified AI chatbots on the predefined frequency and intensity, offering practical insights for researchers who sought to integrate this technology into health behavior change intervention studies. Fourth, we mapped chatbot functionalities onto the health BCT framework to help practitioners select appropriate BCTs for AI chatbots. Finally, we refined the digital validation tools by incorporating engagement measures, providing future intervention studies with clearer guidance on assessing chatbot performance comprehensively.

#### Limitations and Future Studies

Several limitations should be noted. First, we excluded studies that integrated audio-based chatbots, embodied conversational agents, humanoid coach virtual reality, augmented reality virtual coach, therapeutic robots, etc. This occurred because our focus was on the communication characteristics of AI chatbots in health behavior change rather than visual, action, or simulated environments. These additional characteristics add another layer of complexity to the deployment of AI chatbots. Future reviews could explore different AI chatbots that include these technologies. Second, we limited our study to publications in English, which might exclude relevant chatbots developed in other languages. Future reviews could consider including studies published in other languages. We also strongly encourage researchers conducting studies in non-English–speaking countries to publish their findings in English to enable cross-cultural comparisons. Finally, we included all types of studies to provide a more comprehensive synthesis, even though some were of relatively low quality. However, the heterogeneity in study designs and methodologies may limit the comparability of findings and the overall strength of the conclusions. We encourage future systematic reviews and meta-analyses to draw more robust insights by focusing on high-quality studies only.

### Conclusions

This scoping review offers a comprehensive synthesis of AI chatbots as health behavior change interventions. The analysis revealed that PA was mostly targeted. When designing an AI chatbot, it is important to clearly define its roles (ie, routine coach or on-demand assistant or the combination) as well as to specify its theoretical foundation (eg, CBT), BCTs (eg, goals and planning), and technology workflow (eg, Google Dialogflow integrated with Facebook Messenger). The performance of AI chatbots can be evaluated across 5 clusters: technical, health behavior change, usability, engagement, and cost. Future studies should explore more on cost, sleep, weight management, sedentary behavior, and alcohol use to provide more comprehensive evidence. Additionally, they should also examine implementation outcomes to enhance the scalability of AI chatbot interventions. Moreover, rigorous RCTs in diverse populations are needed to generate robust and generalizable findings. Finally, the sustainability of AI chatbot effects on health behavior change along with factors such as intervention duration, modality, and engagement (eg, use duration, frequency, and intensity), as well as the interactions among the 5 evaluation clusters, warrant further exploration.

## Appendix

Multimedia Appendix 1 PRISMA-ScR checklist.

Multimedia Appendix 2 Detailed information on search terms, study eligibility criteria, quality assessment, data extraction items, overview of included studies, study description, characteristics of included studies, theoretical foundation, techniques for developing artificial intelligence chatbots, and efficacy and effectiveness of artificial intelligence chatbots on health behavior change.

## Abbreviations

AI: artificial intelligence
API: application programming interface
BCT: behavior change technique
CBT: cognitive behavioral therapy
MHB: multiple health behavior
MI: motivational interviewing
MMAT: Mixed Methods Appraisal Tool
OHB: one health behavior
OR: odds ratio
PA: physical activity
PRISMA-ScR: Preferred Reporting Items for Systematic Reviews and Meta-Analyses extension for Scoping Reviews
RCT: randomized controlled trial
WAT: wearable activity tracker
