# Supplementary material for: The Development and Use of AI Chatbots for Health Behavior Change: Scoping Review
Source: J Med Internet Res. 2026 Jan 28;28:e79677. doi: 10.2196/79677 (PMC12895150; doi:10.2196/79677)
Supplement: Multimedia Appendix 2 [file jmir_v28i1e79677_app2.docx]

**Table S1.** Search terms by database.

| ***PubMed*** | |
| --- | --- |
| **Search date** | 13 March, 2024 |
| **Search terms** | lifestyle*[tiab] OR tobac*[tiab] OR cigarette*[tiab] OR cigar* [tiab] OR vap*[tiab] OR smok*[tiab] OR nico*[tiab] OR sleep*[tiab] OR bedtime[tiab] OR nap[tiab] OR insomnia[tiab] OR physical activ*[tiab] OR sport*[tiab] OR exercise*[tiab] OR diet*[tiab] OR nutriti*[tiab] OR eating [tiab] OR food*[tiab] OR appetite*[tiab] OR *weight*[tiab] OR obes*[tiab] OR sedentar*[tiab] OR screen time [tiab] OR stress* [tiab] |
|  | AND (Chatbot*[tiab] OR chat-bot*[tiab] OR chat bot*[tiab] OR chat robot*[tiab] OR virtual robot*[tiab] OR voice-bot[tiab] OR social bot*[tiab] OR social robot*[tiab] OR infobot*[tiab] OR health bot*[tiab] OR smartbot*[tiab] OR conversational bot*[tiab] OR artificial intelligence chatbot*[tiab] OR Ai agent*[tiab] OR conversational agent*[tiab] OR dialogue agent*[tiab] OR dialog agent* [tiab] OR interactive agent*[tiab] OR virtual agent*[tiab] OR automated agent*[tiab] OR relational agent*[tiab] OR AI assist*[tiab] OR conversational assistant*[tiab] OR digital assist*[tiab] OR intelligent assist*[tiab] OR virtual assist*[tiab] OR smart assist*[tiab] OR voice assist*[tiab] OR speech assist*[tiab] OR virtual health assist*[tiab] OR dialogue agent*[tiab] OR dialog agent*[tiab] OR AI advisor*[tiab] OR virtual advisor*[tiab] OR animated advisor*[tiab] OR smart advisor*[tiab] OR AI avatar*[tiab] OR virtual avatar*[tiab] OR animated avatar*[tiab] OR smart avatar*[tiab] OR AI coach*[tiab] OR virtual coach*[tiab] OR smart coach*[tiab] OR animated coach*[tiab] OR artificial conversation entit*[tiab] OR Assistance technolog*[tiab] OR conversational AI[tiab] OR conversational interface*[tiab] OR conversational system*[tiab] OR Dialog system*[tiab] OR dialogue system*[tiab] OR natural language interface*[tiab] OR automated conversation[tiab] OR virtual conversation[tiab] OR chatGPT[tiab]) |
| **Filtering** | Include eng[la] |
|  | Not include Systematic review[pt] OR meta-analysis[pt] OR review[pt] |
| **Numbers** | *N* (original) = 1,514  *N* (filter language) = 1,482  N (filter article type) = 1,235 |
| **Note** | Search by title and abstract [tiab]  Language type [la]  Publication type[pt] |
| ***CINAHL (Cumulative Index to Nursing and Allied Health Literature)*** | |
| **Search date** | 13 March, 2024 |
| **Search terms** | AB (lifestyle* OR tobac* OR cigarette* OR cigar* OR vap* OR smok* OR nico* OR sleep* OR bedtime OR nap OR insomnia OR "physical activ*" OR sport* OR exercise* OR diet* OR nutriti* OR eating OR food* OR appetite* OR weight* OR obes* OR sedentar* OR "screen time" OR stress*) |
|  | AB (Chatbot* OR chat-bot* OR "chat bot*" OR "chat robot*" OR "virtual robot*" OR voice-bot OR "social bot*" OR "social robot*" OR infobot* OR "health bot*" OR smartbot* OR "conversational bot*" OR "artificial intelligence chatbot*" OR "Ai agent*" OR "conversational agent*" OR "dialogue agent*" OR "dialog agent*" OR "interactive agent*" OR "virtual agent*" OR "automated agent*" OR "relational agent*" OR "AI assist*" OR "virtual assist*" OR "conversational assistant*" OR "digital assist*" OR "intelligent assist*" OR "virtual assist*" OR "smart assist*" OR "voice assist*" OR "speech assist*" OR "virtual health assist*" OR "dialogue agent*" OR "dialog agent*" OR "AI advisor*" OR "virtual advisor*" OR "animated advisor*" OR "smart advisor*" OR "AI avatar*" OR "virtual avatar*" OR "animated avatar*" OR "smart avatar*" OR "AI coach*" OR "virtual coach*" OR "smart coach*" OR "animated coach*" OR "artificial conversation entit*" OR "Assistance technolog*" OR "conversational AI" OR "conversational interface*" OR "conversational system*" OR "Dialog system*" OR "dialogue system*" OR "natural language interface*" OR "automated conversation" OR "virtual conversation" OR chatGPT) |
| **Filtering** | Include LA English only |
|  | NOT include PT (systematic review or meta-analysis or meta- analysis or scoping review or review) |
|  | Limited source type: academic article |
| **Numbers** | N (original) = 450  N (filter language) = 447  N (filter publication type) = 386  N (filter sources type) = 370 |
| **Note** | Search by abstract (AB) |
| ***Medline*** | |
| **Search date** | 13 March, 2024 |
| **Search terms** | AB (lifestyle* OR tobac* OR cigarette* OR cigar* OR vap* OR smok* OR nico* OR sleep* OR bedtime OR nap OR insomnia OR "physical activ*" OR sport* OR exercise* OR diet* OR nutriti* OR eating OR food* OR appetite* OR weight* OR obes* OR sedentar* OR "screen time" OR stress*) |
|  | AB (Chatbot* OR chat-bot* OR "chat bot*" OR "chat robot*" OR "virtual robot*" OR voice-bot OR "social bot*" OR "social robot*" OR infobot* OR "health bot*" OR smartbot* OR "conversational bot*" OR "artificial intelligence chatbot*" OR "Ai agent*" OR "conversational agent*" OR "dialogue agent*" OR "dialog agent*" OR "interactive agent*" OR "virtual agent*" OR "automated agent*" OR "relational agent*" OR "AI assist*" OR "virtual assist*" OR "conversational assistant*" OR "digital assist*" OR "intelligent assist*" OR "virtual assist*" OR "smart assist*" OR "voice assist*" OR "speech assist*" OR "virtual health assist*" OR "dialogue agent*" OR "dialog agent*" OR "AI advisor*" OR "virtual advisor*" OR "animated advisor*" OR "smart advisor*" OR "AI avatar*" OR "virtual avatar*" OR "animated avatar*" OR "smart avatar*" OR "AI coach*" OR "virtual coach*" OR "smart coach*" OR "animated coach*" OR "artificial conversation entit*" OR "Assistance technolog*" OR "conversational AI" OR "conversational interface*" OR "conversational system*" OR "Dialog system*" OR "dialogue system*" OR "natural language interface*" OR "automated conversation" OR "virtual conversation" OR chatGPT) |
| **Filtering** | Limited by LA english |
|  | Not include PT (systematic review or meta-analysis or meta- analysis or scoping review or review) |
|  | Limited source type: academic article |
| **Numbers** | N (original) = 1,357  N (filter language) = 1,329  N (filter publication type) = 1,108  N (filter sources type) = 1,108 |
| **Note** | Search by abstract (AB) |
| ***EMBASE (Excerpta Medica dataBASE)*** | |
| **Search date** | 13 March, 2024 |
| **Search terms** | ((lifestyle* OR tobac* OR cigarette* OR cigar* OR vap* OR smok* OR nico* OR sleep* OR bedtime OR nap OR insomnia OR 'physical activ*' OR sport* OR exercise* OR diet* OR nutriti* OR eating OR food* OR appetite* OR weight* OR obes* OR sedentar* OR 'screen time' OR 'stress'):ab) |
|  | AND ((Chatbot* OR 'chat-bot*' OR 'chat bot*' OR 'chat robot*' OR 'virtual robot*' OR voice-bot OR 'social bot*' OR 'social robot*' OR 'infobot*' OR 'health bot*' OR 'smartbot*' OR 'conversational bot*' OR 'artificial intelligence chatbot*' OR 'Ai agent*' OR 'conversational agent*' OR 'dialogue agent*' OR 'dialog agent*' OR 'interactive agent*' OR 'virtual agent*' OR 'automated agent*' OR 'relational agent*' OR 'AI assist*' OR 'virtual assist*' OR 'conversational assistant*' OR 'digital assist*' OR 'intelligent assist*' OR 'virtual assist*' OR 'smart assist*' OR 'voice assist*' OR 'speech assist*' OR 'virtual health assist*' OR 'dialogue agent*' OR 'dialog agent*' OR 'AI advisor*' OR 'virtual advisor*' OR 'animated advisor*' OR 'smart advisor*' OR 'AI avatar*' OR 'virtual avatar*' OR 'animated avatar*' OR 'smart avatar*' OR 'AI coach*' OR 'virtual coach*' OR 'smart coach*' OR 'animated coach*' OR 'artificial conversation entit*' OR 'Assistance technolog*' OR 'conversational AI' OR 'conversational interface*' OR 'conversational system*' OR 'Dialog system*' OR 'dialogue system*' OR 'natural language interface*' OR 'automated conversation' OR 'virtual conversation' OR chatGPT):ab) |
| **Filtering** | AND ((english):la) |
|  | AND ((article OR 'article in press' OR 'conference paper'):it) |
| **Numbers** | N (original) = 1,583  N (filter language) = 1,540  N (filter publication type) = 910 |
| **Note** | Search by Abstract (ab)  Language (la)  Publication type (it) |
| ***Web of Science*** | |
| **Search date** | 13 March, 2024 |
| **Search terms** | AB = (lifestyle* OR tobac* OR cigarette* OR cigar* OR vap* OR smok* OR nico* OR sleep* OR bedtime OR nap OR insomnia OR "physical activ*" OR sport* OR exercise* OR diet* OR nutriti* OR eating OR food* OR appetite* OR weight* OR obes* OR sedentar* OR "screen time" OR stress*) |
|  | and AB=(Chatbot* OR chat-bot* OR "chat bot*" OR "chat robot*" OR "virtual robot*" OR voice-bot OR "social bot*" OR "social robot*" OR infobot* OR "health bot*" OR smartbot* OR "conversational bot*" OR "artificial intelligence chatbot*" OR "Ai agent*" OR "conversational agent*" OR "dialogue agent*" OR "dialog agent*" OR "interactive agent*" OR "virtual agent*" OR "automated agent*" OR "relational agent*" OR "AI assist*" OR "virtual assist*" OR "conversational assistant*" OR "digital assist*" OR "intelligent assist*" OR "virtual assist*" OR "smart assist*" OR "voice assist*" OR "speech assist*" OR "virtual health assist*" OR "dialogue agent*" OR "dialog agent*" OR "AI advisor*" OR "virtual advisor*" OR "animated advisor*" OR "smart advisor*" OR "AI avatar*" OR "virtual avatar*" OR "animated avatar*" OR "smart avatar*" OR "AI coach*" OR "virtual coach*" OR "smart coach*" OR "animated coach*" OR "artificial conversation entit*" OR "Assistance technolog*" OR "conversational AI" OR "conversational interface*" OR "conversational system*" OR "Dialog system*" OR "dialogue system*" OR "natural language interface*" OR "automated conversation" OR "virtual conversation" OR chatGPT ) |
| **Filtering** | and English (Languages) |
|  | and Article or Early Access or Proceeding Paper or Correction (Document Types) |
| **Numbers** | N (original) = 1,755  N (filter language) = 1,717  N (filter article type) = 1,504 |
| **Note** | Search by Abstract (AB)  Early access: An article that has been electronically published by a journal before it has been assigned to a specific volume and issue.  Proceedings paper: Full papers in a wide range of disciplines that were or will be presented at a symposium or meeting. The papers to be included must have been presented in full at a conference, meeting, symposium or similar gathering. Generally published in a book of conference proceedings. |
| ***Scopus (scientific, technical, medical and social sciences literature)*** | |
| **Search date** | 13 March, 2024 |
| **Search terms** | ABS(lifestyle* OR tobac* OR cigarette* OR cigar* OR vap* OR smok* OR nico* OR sleep* OR bedtime OR nap OR insomnia OR "physical activ*" OR sport* OR exercise* OR diet* OR nutriti* OR eating OR food* OR appetite* OR *weight* OR obes* OR sedentar* OR "screen time" OR stress*) |
|  | AND ABS(Chatbot* OR chat-bot* OR "chat bot*" OR "chat robot*" OR "virtual robot*" OR voice-bot OR "social bot*" OR "social robot*" OR infobot* OR "health bot*" OR smartbot* OR "conversational bot*" OR "artificial intelligence chatbot*" OR "Ai agent*" OR "conversational agent*" OR "dialogue agent*" OR "dialog agent*" OR "interactive agent*" OR "virtual agent*" OR "automated agent*" OR "relational agent*" OR "AI assist*" OR "virtual assist*" OR "conversational assistant*" OR "digital assist*" OR "intelligent assist*" OR "virtual assist*" OR "smart assist*" OR "voice assist*" OR "speech assist*" OR "virtual health assist*" OR "dialogue agent*" OR "dialog agent*" OR "AI advisor*" OR "virtual advisor*" OR "animated advisor*" OR "smart advisor*" OR "AI avatar*" OR "virtual avatar*" OR "animated avatar*" OR "smart avatar*" OR "AI coach*" OR "virtual coach*" OR "smart coach*" OR "animated coach*" OR "artificial conversation entit*" OR "Assistance technolog*" OR "conversational AI" OR "conversational interface*" OR "conversational system*" OR "Dialog system*" OR "dialogue system*" OR "natural language interface*" OR "automated conversation" OR "virtual conversation" OR chatGPT) |
| **Filtering** | AND ( LIMIT-TO ( LANGUAGE,"English" ) ) |
|  | AND ( LIMIT-TO ( DOCTYPE,"ar" ) OR LIMIT-TO ( DOCTYPE,"cp" ) ) |
| **Numbers** | N (original) = 4,475  N (filter language) = 4,346  N (filter article type) = 3,516 |
| **Note** | Search by Abstract (ABS)  "ar": article  "cp": conference paper |
| ***APA PsycINFO (databased produced by APA)*** | |
| **Search date** | 13 March, 2024 |
| **Search terms** | AB (lifestyle* OR tobac* OR cigarette* OR cigar* OR vap* OR smok* OR nico* OR sleep* OR bedtime OR nap OR insomnia OR "physical activ*" OR sport* OR exercise* OR diet* OR nutriti* OR eating OR food* OR appetite* OR weight* OR obes* OR sedentar* OR "screen time" OR stress*) |
|  | AB (Chatbot* OR chat-bot* OR "chat bot*" OR "chat robot*" OR "virtual robot*" OR voice-bot OR "social bot*" OR "social robot*" OR infobot* OR "health bot*" OR smartbot* OR "conversational bot*" OR "artificial intelligence chatbot*" OR "Ai agent*" OR "conversational agent*" OR "dialogue agent*" OR "dialog agent*" OR "interactive agent*" OR "virtual agent*" OR "automated agent*" OR "relational agent*" OR "AI assist*" OR "virtual assist*" OR "conversational assistant*" OR "digital assist*" OR "intelligent assist*" OR "virtual assist*" OR "smart assist*" OR "voice assist*" OR "speech assist*" OR "virtual health assist*" OR "dialogue agent*" OR "dialog agent*" OR "AI advisor*" OR "virtual advisor*" OR "animated advisor*" OR "smart advisor*" OR "AI avatar*" OR "virtual avatar*" OR "animated avatar*" OR "smart avatar*" OR "AI coach*" OR "virtual coach*" OR "smart coach*" OR "animated coach*" OR "artificial conversation entit*" OR "Assistance technolog*" OR "conversational AI" OR "conversational interface*" OR "conversational system*" OR "Dialog system*" OR "dialogue system*" OR "natural language interface*" OR "automated conversation" OR "virtual conversation" OR chatGPT) |
| **Filtering** | Limited by LA English |
|  | Not include PT (systematic review or meta-analysis or meta- analysis or scoping review or review) |
|  | Limited source type: academic article |
| **Number** | N (original) = 544  N (filter language) = 527  N (filter publication type) = 527  N (filter sources type) = 424 |
| **Note** | Search by Abstract (AB) |
| ***IEEE Xplore*** | |
| **Search date** | 13 March, 2024 |
| **Search terms** | lifestyle OR lifestyles OR tobac* OR cigarette OR cigarettes OR cigar* OR vap OR smok* OR nicotine OR sleep* OR bedtime OR nap OR insomnia OR "physical activ*" OR sport OR sports OR exercise OR exercises OR diet* OR nutri* OR eating OR food OR appetite OR appetites OR *weight* OR sedentary OR sedentaries OR "screen time" OR stress OR stressed |
|  | AND (Chatbot* OR chat-bot OR chat-bots OR "chat bot" OR "chat bots" OR "chat robot" OR "chat robots" OR "virtual robot" OR "virtual robots" OR voice-bot OR voice-bots OR "social bot" OR "social bots" OR "social robot" OR "social robots" OR infobot OR infobots OR "health bot" OR "health bots" OR smartbot OR smartbots OR "conversational bot" OR "conversational bots" OR "artificial intelligence chatbot" OR "artificial intelligence chatbots" OR "Ai agent" OR "Ai agents" OR "conversational agent" OR "conversational agents" OR "dialogue agent" OR "dialogue agents" OR "dialog agent" OR "dialog agents" OR "interactive agent" OR "interactive agents" OR "virtual agent" OR "virtual agents" OR "automated agent" OR "automated agents" OR "relational agent" OR "relational agents" OR "AI assistance" OR "AI assistant" OR "AI assistants" OR "conversational assistant" OR "conversational assistants" OR "digital assist" OR "digital assistant" OR "digital assistants" OR "digital assistance" OR "intelligent assist" OR "intelligent assistant" OR "intelligent assistants" OR "intelligent assistance" OR "virtual assist" OR "virtual assistance" OR "virtual assistant" OR "virtual assistants" OR "smart assist" OR "smart assistance" OR "smart assistant" OR "smart assistants" OR "voice assist" OR "voice assistant" OR "voice assistants" OR "speech assistance" OR "dialogue agent" OR "dialogue agents" OR "dialog agent" OR "dialog agents" OR "AI advisor" OR "AI advisors" OR "virtual advisor" OR "virtual advisors" OR "smart advisor" OR "smart advisors" OR "AI avatar" OR "AI avatars" OR "virtual avatar" OR "virtual avatars" OR "animated avatar" OR "animated avatars" OR "smart avatar" OR "smart avatars" OR "AI coach" OR "AI coaches" OR "virtual coach" OR "virtual coaches" OR "smart coach" OR "smart coaches" OR "Assistance technologies" OR "conversational AI" OR "conversational interface" OR "conversational interfaces" OR "conversational system" OR "conversational systems" OR "Dialog system" OR "Dialog systems" OR "dialogue system" OR "dialogue systems" OR "natural language interface" OR "natural language interfaces" OR "automated conversation" OR "automated conversations" OR "virtual conversation" OR "virtual conversations" OR chatGPT) |
| **Filtering** | break down by “Conferences” and “Journals” and “Early Access Articles” |
| **Numbers** | N (original) = 637  N (filter article type) = 621 |
| **Note** | Search by abstract  Limit the toftal number of wildcards to 9. So, we deleted many wildcards of the terms used in searching other database |
| ***ACM Digital Library*** | |
| **Search date** | 13 March, 2024 |
| **Search terms** | Abstract:(lifestyle* OR tobac* OR cigarette* OR cigar* OR vap* OR smok* OR nico* OR sleep* OR bedtime OR nap OR insomnia OR "physical activ*" OR sport* OR exercise* OR diet* OR nutriti* OR eating OR food* OR appetite* OR weight* OR obes* OR sedentar* OR "screen time" OR stress*) |
|  | Abstract:(Chatbot* OR chat-bot* OR "chat bot*" OR "chat robot*" OR "virtual robot*" OR voice-bot OR "social bot*" OR "social robot*" OR infobot* OR "health bot*" OR smartbot* OR "conversational bot*" OR "artificial intelligence chatbot*" OR "Ai agent*" OR "conversational agent*" OR "dialogue agent*" OR "dialog agent*" OR "interactive agent*" OR "virtual agent*" OR "automated agent*" OR "relational agent*" OR "AI assist*" OR "virtual assist*" OR "conversational assistant*" OR "digital assist*" OR "intelligent assist*" OR "virtual assist*" OR "smart assist*" OR "voice assist*" OR "speech assist*" OR "virtual health assist*" OR "dialogue agent*" OR "dialog agent*" OR "AI advisor*" OR "virtual advisor*" OR "animated advisor*" OR "smart advisor*" OR "AI avatar*" OR "virtual avatar*" OR "animated avatar*" OR "smart avatar*" OR "AI coach*" OR "virtual coach*" OR "smart coach*" OR "animated coach*" OR "artificial conversation entit*" OR "Assistance technolog*" OR "conversational AI" OR "conversational interface*" OR "conversational system*" OR "Dialog system*" OR "dialogue system*" OR "natural language interface*" OR "automated conversation" OR "virtual conversation" OR chatGPT) |
| **Filtering** | break down by “Research Article” and “Short paper” |
| **Numbers** | N (original) =1,176  N (filter article type) = 820 |
| **Note** | Search by abstract |

**Table S2.** Study eligibility criteria.

| **Category** | **Subcategory** | **Inclusion criteria** | **Exclusion criteria** |
| --- | --- | --- | --- |
| ***Study characteristics*** | | | |
| P (Population) | Sex, age, species | Population of any gender, age, and ethnicity. | None |
| I (Intervention) | Location | Studies in any country. | None |
|  | Technique | AI chatbot: Chatbot developed upon existing AI platforms (e.g., IBM Watson), machine learning algorithms (e.g., Recurrent Neural Network), and natural language process (e.g., sentiment analysis). | Non-AI chatbot: Chatbot developed upon non-AI algorithm (e.g., rule-based algorithms, pattern-matching algorithm, RiveScript, Chatscript). |
| C (Comparison) | None | With or without dependent comparison (e.g., pre-post comparison) and independent comparison (e.g., AI chatbot vs. control group/non-AI chatbot; text-based AI chatbot vs. audio-based AI chatbot). | None |
| O (Outcomes) | None | Physical activity, diet, sleep, weight management, sedentary behavior, stress management, smoking, and alcohol consumption. | Other behaviors. |
| S (Study types) | None | Peer-reviewed empirical studies with either qualitative or quantitative data published by scholarly journals. | Review papers (e.g., systematic review, scoping review, meta-analysis), observational study (e.g., cross-sectional, cohort, case-control), protocols, editorials, opinion pieces, conference abstracts, and dissertations. |
| ***Publication characteristics*** | | | |
| P (Publication) | Publication dates | Before March 2024 | None |
|  | Language | English | Other Language |
|  | Publication status | Full online | None |

**Table S3.** Study quality assessment [16–20,23,24,33–68].

| **Articles** | **Screening** | | **1. Qualitative (QS)** | | | | |
| --- | --- | --- | --- | --- | --- | --- | --- |
|  | S1 | S2 | 1.1. Is the qualitative approach appropriate to answer the research question? | 1.2. Are the qualitative data collection methods adequate to address the research question? | 1.3. Are the findings adequately derived from the data? | 1.4. Is the interpretation of results sufficiently substantiated by data? | 1.5. Is there coherence between qualitative data sources, collection, analysis and interpretation? |
| Figueroa et al. (2021) [16] | Yes | Yes | Yes | Yes | Yes | Yes | Yes |
| Aarts et al. (2022) [61] | Yes | Yes | Yes | Yes | Yes | Yes | Yes |
| **Articles** | **Screening** | | **2. Quantitative randomized controlled trials (RCTs)** | | | | |
|  | S1 | S2 | 2.1. Is randomization appropriately performed? | 2.2. Are the groups comparable at baseline? | 2.3. Are there complete outcome data? | 2.4. Are outcome assessors blinded to the intervention provided? | 2.5 Did the participants adhere to the assigned intervention? |
| Albers, Hizli et al. (2023) [47] | Yes | Yes | Yes | Yes | Yes | Can't tell | Yes |
| Alghamdi et al. (2021) [36] | Yes | Yes | Can't tell | Yes | Yes | No | Yes |
| Catellani et al. (2023) [37] | Yes | Yes | Yes | Yes | Yes | Yes | Yes |
| Danieli et al. (2021) [50] | Yes | Yes | Can't tell | Yes | Yes | No | Yes |
| Danieli et al. (2022) [51] | Yes | Yes | Yes | No | No | No | Yes |
| Fitzsimmons‐Craft et al. (2022) [39] | Yes | Yes | Yes | Yes | No | No | No |
| Hassoon et al. (2021) [38] | Yes | Yes | Yes | Yes | Yes | Yes | Yes |
| Medeiros et al. (2021) [42] | Yes | Yes | Yes | Yes | No | No | Yes |
| Karhiy et al. (2023) [24] | Yes | Yes | Yes | Yes | Yes | No | Yes |
| Meng & Dai (2021) [23] | Yes | Yes | Yes | No | No | No | Yes |
| Olano-Espinosa et al. (2022) [19] | Yes | Yes | Yes | Yes | No | Yes | No |
| Perski et al. (2019) [34] | Yes | Yes | Yes | No | Yes | No | No |
| Piao, Ryu et al. (2020) [58] | Yes | Yes | Yes | Yes | Yes | No | Yes |
| Carrasco-Hernandez et al. (2020) [46] | Yes | Yes | Yes | Yes | No | Yes | No |
| **Articles** | **Screening** | | **3. Quantitative nonrandomized (Non-RCTs)** | | | | |
|  | S1 | S2 | 3.1. Are the participants representative of the target population? | 3.2. Are measurements appropriate regarding both the outcome and intervention (or exposure)? | 3.3. Are there complete outcome data? | 3.4. Are the confounders accounted for in the design and analysis? | 3.5. During the study period, is the intervention administered (or exposure occurred) as intended? |
| Albino de Queiroz et al. (2023) [35] | Yes | Yes | No | Yes | Yes | No | Yes |
| Brown et al. (2023) [88] | Yes | Yes | Yes | Yes | Yes | No | Yes |
| Daley et al. (2020) [52] | Yes | Yes | No | Yes | Yes | No | Yes |
| Davis et al. (2020) [49] | Yes | Yes | No | Yes | Yes | No | Yes |
| Dhinagaran et al (2021) [67] | Yes | Yes | No | Yes | Yes | No | Yes |
| Maher et al. (2020) [48] | Yes | Yes | No | Yes | Yes | No | Yes |
| Sia et al. (2021) [66] | Yes | Yes | No | No | No | No | No |
| Sun et al. (2023) [68] | Yes | Yes | No | Can't tell | Yes | Yes | Yes |
| To et al. (2021) [20] | Yes | Yes | No | Yes | Yes | Yes | Yes |
| Durden et al. (2023) [54] | Yes | Yes | No | Yes | Yes | No | Yes |
| De Nieva et al. (2020) [53] | Yes | Yes | No | Can't tell | No | No | Yes |
| Forman-Hoffman et al. (2023) [55] | Yes | Yes | No | Yes | Yes | No | Yes |
| Hoffman et al. (2023) [56] | Yes | Yes | No | Yes | Yes | No | Yes |
| Prochaska et al. (2021) [62] | Yes | Yes | No | Yes | No | No | Yes |
| Masaki et al. (2019) [45] | Yes | Yes | No | Yes | Yes | No | Yes |
| **Articles** | **Screening** | | **4. Quantitative descriptive (QD)** | | | | |
|  | S1 | S2 | 4.1. Is the sampling strategy relevant to address the research question? | 4.2. Is the sample representative of the target population? | 4.3. Are the measurements appropriate? | 4.4. Is the risk of nonresponse bias low? | 4.5. Is the statistical analysis appropriate to answer the research question? |
| Almusharraf et al. (2020) [59] | Yes | Yes | Yes | No | Yes | Yes | Yes |
| Cameron et al. (2018) [33] | Yes | Yes | Yes | No | Yes | Yes | Yes |
| Fadhil et al. (2019) [65] | Yes | Yes | Yes | No | Yes | Yes | Yes |
| Larizza et al. (2023) [40] | Yes | Yes | Yes | No | No | Yes | Yes |
| Piao, Kim et al. (2020) [44] | Yes | Yes | Yes | No | Can't tell | Yes | Yes |
| Stephens et al. (2019) [41] | Yes | Yes | Yes | No | Yes | No | Yes |
| Holmes et al. (2019) [17] | Yes | Yes | Yes | No | Yes | Yes | Yes |
| Griol et al. (2022) [63] | Yes | Yes | Yes | No | Yes | Yes | Yes |
| **Articles** | **Screening** | | **5. Mixed methods (MMS)** | | | | |
|  | S1 | S2 | 5.1. Is there an adequate rationale for using a mixed methods design to address the research question? | 5.2. Are the different components of the study effectively integrated to answer the research question? | 5.3. Are the outputs of the integration of qualitative and quantitative components adequately interpreted? | 5.4. Are divergences and inconsistencies between quantitative and qualitative results adequately addressed? | 5.5. Do the different components of the study adhere to the quality criteria of each tradition of the methods involved? |
| Legaspi et al. (2022) [18] | Yes | Yes | Yes | Yes | Yes | Yes | Yes |
| Moore et al. (2024) [57] | Yes | Yes | Yes | Yes | Yes | Yes | Yes |
| Rahmanti et al. (2020) [64] | Yes | Yes | Yes | Yes | Yes | Yes | Yes |
| Albers, Neerincx et al. (2023) [43] | Yes | Yes | Yes | Yes | Yes | Yes | Yes |

***Note.* S1.** Are there clear research questions? **S2.** Do the collected data allow us to address the research questions?

**'Yes'** means that the criterion is met; **‘No'** means that the criterion is not met; **'Can't tell'** means that there is not enough information in the paper for you to judge if the criterion is met or not.

**Table S4.** Data extraction items.

| **Publication** | **Description** |
| --- | --- |
| Author | The first and corresponding author |
| Year | Publication year |
| Journal/Conference | Name of the journal or conference |
| Country | Corresponding country |
| **Exposure and Outcome** |  |
| Targeted behaviors | Physical activity, diet, sleep, weight management, sedentary behavior, stress management, smoking, and alcohol consumption. |
| Chatbot demographic information | Name, gender, language |
| Theoretical foundations | What theories (e.g., social cognitive therapy) are used for designing AI chatbots? |
| Health behavior changes function | What specific functions or features have been designed to fit the role? |
| Technologies | What technologies were used to develop AI chatbots? |
| **Population** |  |
| Target population | Describe the characteristics of the targeted populations, such as overweight, depressed, anxiety, insomnia, anorexia, undergraduate students, working adults, etc. |
| #Participation | Describe the number of participants |
| Age | Describe the average age |
| **Intervention** |  |
| Study type [32] | **Randomized controlled trials (RCTs):** a study in which individual participants are allocated to intervention or control groups by randomization.  **Quantitative nonrandomized trials (Non-RCTs):** any quantitative studies estimating the effectiveness of an intervention or studying other exposures that do not use randomization to allocate units to comparison groups, such as a pre-post study.  **Qualitative study (QS):** qualitative approach for exploring and understanding the meaning individuals or groups ascribe to a social or human problem, such as narrative research.  **Quantitative descriptive study (DS):** a study that only describes the existing distribution of variables without much regard to causal relationships or other hypotheses.  **Mixed methods study (MMD):** Involve combining qualitative and quantitative methods. |
| Intervention description | Does this chatbot work individually or integrate with other interventions? |
| Usage dose | What is the total duration of use, timing of use, frequency of use, and duration per interaction? |
| Intervention period | How long did the intervention last? |
| **AI chatbot performance validation** | |
| Technical | Technical refers to testing if the AI chatbots actually perform to their self-proclaimed functionality with accuracy and precision. |
| Usability | Usability refers to how easy an AI chatbot is to use for its intended purpose and the minimal effort required to complete tasks. |
| Engagement | Engagement with digital behavior change interventions (DBCIs) includes (1) the extent of usage, such as amount, frequency, duration, and depth; (2) a subjective experience characterized by attention, and affect |
| Cost | Cost refers to the price for user access, technology lifecycle expenses, and integration costs within clinical workflows. |
| Health behavior change | Health behavior change refers to whether the AI chatbots impact the predefined health behavior change outcomes. |
| Implementation outcomes | The effects of deliberate and purposive actions to implement AI chatbots, such as reach, adoption, cost-effectiveness, fidelity, maintenance, scalability, and effectiveness. |

**Table S5.** Overview of included studies [16–20,23,24,33–68]

| **Author (year)** | **Country** | **Behaviors** | **Population** | **Study type** | **Study Description** | **Technology [Frontend & Backend & External]** | **Total Duration/timing of use/usage frequency/duration per interaction** | **Outcome** |
| --- | --- | --- | --- | --- | --- | --- | --- | --- |
| Albers, Hizli et al. (2023) [47] | Netherlands | PA | English speakers and did not participate in the earlier data-gathering studies [n = 39, age = 41.55 (16.15)] | RCTs | 2-arm [G1: generic examples of virtual coach; G2: personalized examples of virtual coach] | Non-code platform [Rasa & Rasa & Rasa cloud] | one-time/N.A./one-time/seven minutes | Usability, engagement, self-efficacy and motivation on PA engagement |
| Albino De Queiroz et al. (2023)[35] | Brazil | Diet & PA | Colorectal cancer patients [n = 36, age = 58.2 years] | Non-RCTs | 2-arm [IG: chatbot and wearable devices; CG: traditional monitoring] | Non-code platform [Facebook Messenger & Google Dialogflow & Google Cloud] | 8-week/any/any/N.R. | Technical, usability, engagement, and %people increase in PA and food intake |
| Alghamdi & Alnanih (2021) [36] | Saudi Arabia | Diet | Celiac patients [n = 60, NR] | RCTs | 2-arm [IG: chatbot; CG: without any intervention] | Non-code platform [WhatsApp & Widebot & N.R.] | 12-week/any/any/depend on the answer question | % of people having difficulty in finding gluten-free products |
| Almusharraf et al. (2020) [59] | Canada | Smoke | Adult smoker [n = 121, age = 35.2 (9.8) years] | DS | Chatbot only | Self-design [Web platform & spaCy (NLP framework) & N.R.] | one-time/N.R./one-time/N.R. | Technical, mean interaction time, and subjective experience |
| Brown et al. (2023) [88] | Canada | Smoke | Adult smokers [n = 100, age = 18-60 years] | Non-RCTs | Chatbot only | Self-design [Web platform & GPT-2 XL neural network & Google Firebase] | one-time/N.R./one-time/follow pre-defined communication process | Feel of empathy, readiness to quit, quit attempts, reduction of smoking, and ambivalence change |
| Cameron et al. (2018) [33] | UK | Sleep & Stress | Full-time employees [n = 7, age = 25-44 years] | DS | Chatbot only | Self-design [Inspire Support Hub & Microsoft Bot Framework & MySQL] | one-time/N.R./one-time/less than 40 minutes | Technical and usability |
| Catellani et al. (2023) [37] | Italy | PA | Ntotal = 595; Healthy respondents [age = 29.99 (9.44)]; patients diagnosed with cardiovascular problems [age = 59.85 (15.06)] | RCTs | 4-arm [chatbot with gain, or non-loss, or non-gain, or loss messages] | Mobile app [PsyMe] | 2-week/N.R./14 messages daily/N.R. | Perceived involvement and intention to engage in PA |
| Daley et al. (2020) [52] | UK | Stress | Portuguese-speaking adults [n= 3,629, age ≥ 18 years] | Non-RCTs | Chatbot only | Mobile app [Vitalk app] | 4-week/N.R./4-5 conversation per week/each last ~5 minutes | Engagement and perceived stress |
| Danieli et al. (2021) [50] | Italy | Stress | Aging workers with mild to high distress [n = 21, age = 47.76 (8.07) years] | RCTs | 2-arm [G1: traditional virtual SMT-CBT treatment plus chatbot; G2: SMT-CBT treatment only] | Mobile App [Mobile personal health care agent (m-PHA)] | 8-week/any/any/any | Usability, engagement, and psychological stress |
| Danieli et al. (2022) [51] | Italy | Stress | Aging workers [n = 45, age = 55.58 (5.08) years] | RCTs | 4-arm [G1: traditional virtual SMT-CBT treatment g; G2: traditional virtual SMT-CBT treatment + Chatbot; G3: Chatbot only; G4: Control group without any treatment] | Mobile App [Mobile personal health care agent (m-PHA)] | 8-week/any/any/any | Engagement and psychological stress |
| Davis et al. (2020) [49] | Australia | Diet & PA | Middle-aged and older healthy adults [n = 31, age = 56.2 (8) years] | Non-RCTs | Chatbot only | Non-code platform [Slack & IBM Watson & Slack cloud] | 12-week/N.R./weekly check-in, 24/7 answer question/N.R. | Technical, engagement, physical activity, and diet goals adherence |
| Dhinagaran et al (2021) [67] | Singapore | PA & Diet & Sleep & Stress & sedentary | Singapore citizens or permanent residents [n = 75, age = 33.7 (9.3) years] | Non-RCTs | Chatbot only | Non-code platform [Facebook Messenger & Chatfuel & N.R.] | 4-week/N.R./4 per week, 1 for each behavior/N.R. | Technical, usability, engagement, diet, PA, sleep quality, stress, and sitting time |
| Fadhil et al. (2019) [65] | Italy | PA & Diet & Sleep & Stress | Arabic speaking adults [n = 43, age = 29.8 years] | DS | Chatbot only | Non-code platform [Telegram Bot platform & IBM Watson & N.R.] | 1-week/N.R./N.R./N.R. | Technical, usability, and engagement |
| Figueroa et al. (2021) [16] | USA | PA | Low-income Spanish and English-Speaking Women [n = 18, age = 27-41 years] | QS | 2-arm [G1: simulated chatbot; G2: real chatbot] | Non-code platform [SMS & IBM Watson & MongoDB] | one-time/N.A./one-time/10-20 minutes | Technical, usability, and engagement |
| Fitzsimmons‐Craft et al. (2022) [39] | USA | Diet & weight management | Clinical eating disorders [n = 700, age = 21.08 (3.09) years] | RCTs | 2-arm [IG: interact with chatbot immediately; CG: interact with chatbot 6 months later | Non-code platform [SMS or Facebook Messenger & Cass AI & N.R.] | 4-week/each 2 days/2 conversations per week/each conversation ~10 minutes | Engagement, eating disorder, weight and shape concern, and internalization |
| Hassoon et al. (2021) [38] | USA | PA | Overweight or obese and physically inactive cancer survivors [n = 42, age = 62.1(9.8) years] | RCTs | 3-arm [G1: voice-based chatbot (SmartText); G2: text-based chatbot (MyCoach); G3: no intervention] | Self-design [SmartText: SMS & supervised goal-based model & NR; MyCoach: Alexa voice & unsupervised goal-based model with a reward condition & N.R.] | audio: 4-week/any/any/any; text: 4-week/N.R./3 messages per day/N.R. | Engagement and percentage of change in daily steps |
| Medeiros et al. (2022) [42] | Netherlands | Stress | Convenience healthy adult [n = 210, age = 20-64 years] | RCTs | 3-arm [G1: chatbot with telling generated by computer; G2: chatbot with telling by human; G3: control group] | Non-code platform [Facebook messenger & IBM Watson & MongoDB] | 3-day/N.R./1 time per day/<10 minutes per time | Perceived current stress |
| Larizza et al. (2023) [40] | Italy | PA & Diet & Sleep & Weight management | Obese children [n = 13, age = 9.3 (1.3) years] | DS | Chatbot only | Non-code platform [Alexa & Google Dialogflow & PostgreSqL] | 2-week/N.R./N.R./N.R. | Technical, usability, and engagement |
| Legaspi et al. (2022) [18] | Philippines | Sleep & Stress | Senior High students [n = 18, age = 16-19 years] | MMD | Chatbot only | Mobile App [Wysa] | 1-week/N.R./daily/at least 10 minutes daily | Technical, engagement, and perceived stress |
| Karhiy et al. (2023) [24] | New Zealand | Stress | University students [n = 108, age = 24.87 years] | RCTs | 3-arm [G1: virtual human; G2: human teletherapist; G3: chatbot] | Non-code platform [N.R. & IBM Watson & N.R.] | 4-week/N.R./2 sessions per week/15 minutes per session | Technical, engagement, perceived stress, current stress, and physiological stress |
| Maher et al. (2020) [48] | Australia | Diet & PA | Inactive community-dwelling adults [n = 31, age = 56.2(8) years] | Non-RCTs | Chatbot only | Non-code platform [Slack & IBM Watson & Slack cloud] | 12-week/N.R./weekly check-in, 24/7 answer question/N.R. | Engagement, moderate-to-vigorous PA minutes and diet adherence, body weight, and waist circumference |
| Meng & Dai (2021) [23] | USA | Stress | University students [n = 278, age = 20.4 (2.28) years] | RCTs | 6-arm [2 (source: chatbot vs. human) by 2 (emotional support: yes vs. no) by 2 (reciprocal self-disclosure: yes vs. no)] | Non-code platform [Facebook Messenger & Chatfuel & N.R.] | one-time/N.A./one-time/6 turns | Engagement and perceived stress |
| Moore et al. (2024) [57] | UK | PA | Convenience adolescent [n = 46, age = 11-13 years] | MMD | Chatbot only | Non-code platform [Mindbehind & Google Dialogflow & N.R.] | one-time/N.A./one-time/N.A. | Usability, engagement, and motivation and intention in engaging PA |
| Olano-Espinosa et al. (2022) [19] | Spain | Smoke | Adult smokers from 262 health care centers [n = 513, age = 49.8 (10.82) years] | RCTs | 2-arm: IG: chatbot; CG: usual care by human | Self-design [Telegram & Bayes’ theorem and NLP & N.R.] | 24-week/any/any/any | Engagement and biochemically continuous abstinence |
| Perski et al. (2019) [34] | UK | Smoke | Adult smokers [n = 57,214, age ≥ 18 years] | RCTs | 2-arm [IG: ‘pro’ app and AI chatbot; CG: ‘pro’ app] | Mobile App [Smoke-Free app] | 4-week/N.R./2 per day, anytime on demand support/N.R. | Engagement and self-reported continuous abstinence |
| Piao, Kim et al. (2020) [44] | Korea | PA | Office workers [n = 20, age = 20-59 years] | DS | Chatbot only | Non-code platform [FriendPlus & IBM Watson & N.R.] | 3-week/define by user/daily/N.A. | Usability and engagement |
| Piao, Ryu et al. (2020) [58] | Korea | PA | Office workers [n = 121, age = 20-59 years] | RCTs | 2-arm [IG: chatbot with cues, intrinsic and extrinsic rewards for 12 weeks; CG: chatbot without intrinsic rewards for the first 4 weeks, but added from 5 to 12 weeks] | Non-code platform [FriendPlus & IBM Watson & N.R.] | 12-week/predefined by user/daily/N.R. | Habit strength of PA |
| Rahmanti et al. (2022) [64] | Taiwan, China | Diet & Weight Management | International women Medical University students [n = 10, age = 24-34 years] | MMD | Chatbot only | Non-code platform [LINE & Google Dialogflow & SlimMe] | 1-week/N.R./daily/N.R. | Technical, usability, and engagement |
| Sia et al. (2021) [66] | Philippines | PA, Diet & Sleep & Stress | Grade 12 High students [n = 36, age = 17-18 years] | Non-RCTs | Chatbot only | Non-code platform [Facebook Messenger & Google Dialogflow & N.R.] | 1-week/N.R./daily/N.R. | Technical, engagement, and perceived sleep quality |
| Stephens et al. (2019) [41] | USA | Weight management | Obese youth with at least one comorbid condition [n = 23, age = 15.2 years] | DS | Chatbot only | Non-code platform [SMS, WhatsApp, or Facebook Messenger & Cass & Cass cloud] | 10–12 week/N.R./N.R./N.R. | Engagement and %patients targeting goal progress for weight management |
| Sun et al. (2023)[68] | Netherlands | Stress & PA | Non-clinical population [n = 77, age ≥ 16 years] | Non-RCTs | All participants received both IG [chatbot] and CG [no intervention] | Self-design [Telegram & NLP, generative model, dialogue management model & N.R.] | one-time/N.A./N.A./control conversation & 3 randomly assigned experiment interventions | Technical, usability, belief in PA engagement, and perceived stress |
| To et al. (2021) [20] | Australia | PA | Inactive population [n = 116, age = 49.1 (9.3) years] | Non-RCTs | Chatbot only | Non-code platform [Facebook Messenger & Google Dialogflow & N.R.] | 6-week/participant selected times (proactive); as requested (reactive)/daily (proactive); as requested (reactive)/N.R. | Technical, usability, engagement, %Meeting PA guidelines, PA minutes, steps, and body mass index |
| Albers, Neerincx, et al. (2023) [43] | Netherlands | Smoke | Smokers [n = 500, age = 18-74 years] | MMD | Chatbot only | Self-design [mobile app & reinforcement learning & N.R.] | At least 9-day/2-5 days apart/five sessions/each session about 5 to 8 minutes | Usability, engagement, quitter self-identity, ease of and motivation to do quitting activities |
| Aarts et al. (2022) [61] | Netherlands | Sleep | Middle school students suffering from insomnia [n = 5, age = 10(1.5) years] | QS | Chatbot only | Non-code platform [web-interface & Landbot & N.R.] | 1-week/prefer morning wake up/daily/longer if participant reports sleeping poorly | Technical and engagement |
| Holmes et al. (2019) [17] | UK | Weight management | Healthy adults [n = 30, NR] | DS | Chatbot only | Non-code platform [Facebook Messenger & Google Dialogflow & N.R.] | one-time/N.A./one-time/complete task | Technical, usability, and engagement |
| Griol et al. (2022) [63] | Spain | Diet & PA | Convenience healthy adult [n = 20, age ≥ 0 years] | DS | Chatbot only | Non-code platform [Facebook Messenger & Google Dialogflow & N.R.] | one-time/N.A./one-time/complete task | Technical and engagement |
| Durden et al. (2023) [54] | USA | Stress | Healthy adult [n = 262, age = 39 (13.35)] | Non-RCTs | Chatbot only | Mobile App [Woebot] | 8-week/N.R./N.R./N.R. | Perceived stress |
| De Nieva et al. (2020) [53] | Philippines | Stress | Senior High School students [n = 25, NR] | Non-RCTs | Chatbot only | Mobile App [Woebot] | 2-week/N.R./daily/N.R. | Technical, engagement, and perceived stress |
| Forman-Hoffman et al. (2023) [55] | USA | Stress | Residentially vulnerable populations [n = 255, age ≥ 18 years] | Non-RCTs | Chatbot only | Mobile App [Woebot] | 8-week/N.R./N.R./N.R. | Engagement and perceived stress |
| Hoffman et al. (2023) [56] | USA | Stress | Healthy adult [n = 256, age ≥ 18 years] | Non-RCTs | Chatbot only | Mobile App [Woebot] | 8-week/N.R./N.R./N.R. | Engagement and perceived stress |
| Prochaska et al. (2021) [62] | USA | Alcohol | Substance misuse adults without major health contraindications [n = 101, age = 36.8(10) years] | Non-RCTs | Chatbot only | Mobile App [Woebot] | 8-week/N.R./N.R./N.R. | Usability, engagement, alcohol use disorders, craving and confidence to quit |
| Carrasco-Hernandez et al. (2020) [46] | Spain | PA & Smoke & Weight management | Adult smoker [n= 24 0, age = 50 (10) years] | RCTs | 2-arm [IG: psychopharmacological therapy and digital therapeutic solution; CG: psychopharmacological therapy alone] | Self-design [mobile interface & self-learning algorithm & N.R.] | 48-week/N.R./N.R./N.R. | Technical, usability, engagement, biochemically abstinence rate, nicotine metabolites, self-report PA, and body mass index |
| Masaki et al. (2019 [45]) | Japan | Smoke | Adult smokers [n = 56, age = 43.3(10.5) years] | Non-RCTs | Chatbot only | Mobile App [CureApp Smoking Cessation (CASC)] | 24-week/N.R./20 videos across 12-week/1 to 3 minutes per videos | Engagement, biochemically continuous abstinence rate, withdrawal symptoms, tobacco cravings, and psychological aspects of smoking |

***Note.*** RCTs: randomized controlled trials; Non-RCTs: Quantitative nonrandomized trials; QS: Qualitative study; DS: Quantitative descriptive study; MMD: Mixed methods study. N.A.: Not available; N.R.: Not reported

**Table S6.** Publication description (n = 43) [16–20,23,24,33–68]

| **Characteristics** | **Articles** | **n** | **%** |
| --- | --- | --- | --- |
| **Year** |  |  |  |
| 2024 | [57] | 1 | 2.33% |
| 2023 | [24,35,37,40,43,47,54–56,68,88] | 11 | 25.58% |
| 2022 | [18,19,39,42,51,61,63,64] | 8 | 18.60% |
| 2021 | [16,20,23,36,38,50,62,66,67] | 9 | 20.93% |
| 2020 | [44,46,48,49,52,53,58,59] | 8 | 18.60% |
| 2019 | [17,34,41,45,65] | 5 | 11.63% |
| 2018 | [33] | 1 | 2.33% |
| **Country/Region** |  |  |  |
| US | [16,23,38,39,41,54–56,62] | 9 | 20.93% |
| Netherlands | [42,43,47,61,68] | 5 | 11.63% |
| Italy | [37,40,50,51,65] | 5 | 11.63% |
| UK | [17,33,34,52,57] | 5 | 11.63% |
| Spain | [19,46,63] | 3 | 6.98% |
| Australia | [20,48,49] | 3 | 6.98% |
| Philippines | [18,53,66] | 3 | 6.98% |
| Korea | [44,58] | 2 | 4.65% |
| Canada | [59,88] | 2 | 4.65% |
| Others | Japan [45], Taiwan, China [64], Singapore [67], Saudi Arabia [36], New Zealand [24], Brazil [35] | 6 | 13.95% |
| **Publications** |  |  |  |
| ***Journals*** |  | 33 | 76.74% |
| JMIR mHealth and uHealth | [19,20,45,46,48,58] | 6 | 18.18% |
| JMIR Formative Research | [50,55,57,67] | 4 | 12.12% |
| Journal of Medical Internet Research | [56,59,62] | 3 | 9.09% |
| Frontiers in Digital Health | [16,52] | 2 | 6.06% |
| JMIR Mental Health | [51,88] | 2 | 6.06% |
| Others | [23,34–44,47,49,54,64] | 16 | 48.48% |
| ***Conference*** | [17,18,24,33,53,61,63,65,66,68] | 10 | 23.26% |

***Note.*** Others: Journal of Medical Systems [47], Healthcare Analytics [35], International Journal of Advanced Computer Science and Applications (IJACSA) [36], Frontiers in Psychology [37], International Journal of Environment and Public health [49], International Journal of Eating disorders [39], Digital Medicine [38], IEEE Transactions on Human-Machine Systems [42], International Journal of Medical Informatics [40], Journal of Computer-Mediated Communication [23], Digital Health [34], Healthcare informatics research [44], Frontiers in Nutrition [64], Translational Behavioural Medicine [41], Persuasive technology [41], Internet Interventions [54].

**Table S7.** Characteristics of included studies (n = 43) [16–20,23,24,33–68]

| **Items** | **Articles** | **n** | **%** |
| --- | --- | --- | --- |
| **Study type** |  |  |  |
| Randomized controlled trials (RCTs) | [19,23,24,34,36–39,42,46,47,50,51,58] | 14 | 31.82% |
| Quantitative nonrandomized trials (Non-RCTs) | [20,35,45,48,49,52–56,62,66–68,88] | 15 | 34.09% |
| Quantitative descriptive study (DS) | [17,33,40,41,44,59,63,65] | 8 | 18.18% |
| Qualitative study (QS) | [16,61] | 2 | 4.55% |
| Mixed methods study (MMD) | [18,43,57,64] | 4 | 9.09% |
| **Participants Characteristics** |  |  |  |
| *Non-Clinical (36/43, 83.72%)* |  |  |  |
| Convenient healthy population | [17,42,49,54,56,63,68] | 7 | 16.28% |
| Physically inactive and not a Mediterranean dietary pattern | [20,48] | 2 | 4.65% |
| Smoker | [19,34,43,45,46,59,88] | 7 | 16.28% |
| Substance misuse | [62] | 1 | 2.33% |
| Worker | [33,44,50,51,58] | 5 | 11.63% |
| Middle-to-high school students | [18,53,61,66] | 4 | 9.30% |
| University students | [23,24,64] | 3 | 6.98% |
| Vulnerable populations (low-income) | [16,55] | 2 | 4.65% |
| Others | [47,52,57,65,67] | 5 | 11.63% |
| *Clinic (7/43, 16.28%)* |  |  |  |
| Colorectal cancer patients | [35] | 1 | 2.33% |
| Celiac patients | [36] | 1 | 2.33% |
| Cardiovascular disease patients | [37] | 1 | 2.33% |
| Clinical eating disorder | [39] | 1 | 2.33% |
| Overweight or obese and physically inactive cancer survivors | [38] | 1 | 2.33% |
| Obesity | Obese children [40] and Obese youth [41] | 2 | 4.65% |
| **Age** |  |  |  |
| Middle Childhood (6–11 years) | [40,61] | 2 | 4.65% |
| Early Adolescence (12–18 years) | [18,41,57,66] | 4 | 9.30% |
| Young Adulthood (19–44 years) | [16,23,24,33,37,39,45,47,54,59,62,64,65,67] | 14 | 32.56% |
| Middle Adulthood (45–64 years) | [19,20,35,38,46,48–51] | 9 | 20.93% |
| Across at least three age groups (16-80 years) | [34,42–44,52,55,56,58,63,68,88] | 11 | 25.58% |
| NR | [17,36,53] | 3 | 6.98% |
| **Chatbot usage duration** |  |  |  |
| One-time | [16,17,23,33,47,57,59,63,68,88] | 10 | 23.26% |
| 1-week | [18,61,64–66] | 5 | 11.63% |
| 2-week | [37,40,53] | 3 | 6.98% |
| 4-week | [24,34,38,39,52,67] | 6 | 13.95% |
| 8-week | [35,50,51,54–56,62] | 7 | 16.28% |
| 12-week | [36,41,48,49,58] | 5 | 11.63% |
| Others | 3-day [42], 9-day [43], 3-week [44], 6-week [20], 24-week [19,45], 48-week [46] | 7 | 16.28% |
| **#participants** |  |  |  |
| 7–50 | [16–18,33,35,38,40,41,44,47–51,53,57,61,63–66] | 21 | 48.84% |
| 51–100 | [36,45,67,68,88] | 5 | 11.63% |
| 101–200 | [20,24,58,59,62] | 5 | 11.63% |
| 201–300 | [23,42,46,54–56] | 6 | 13.95% |
| 301–600 | [19,37,43] | 3 | 6.98% |
| Others | 700 [39]; 3,629 [52]; 57,214 [34] | 3 | 6.98% |
| **Language of the Chatbot** |  |  |  |
| English | [16,24,34,42,43,45,47,53–56,59,62,64,68,88] | 16 | 37.21% |
| Arabic | [36,65] | 2 | 4.65% |
| Korean | [44,58] | 2 | 4.65% |
| Spanish | [46] | 1 | 2.33% |
| Portuguese | [52] | 1 | 2.33% |
| NR | [17–20,23,33,35,37–41,48–51,57,61,63,66,67] | 21 | 48.84% |

**Table S8.** Theoretical foundation (n = 43) [16–20,23,24,33–68]

| **Theory** | **Articles** | **n** | **%** |
| --- | --- | --- | --- |
| ***One theory (20/43, 46.51%)*** |  |  |  |
| **Cognitive Behavior Therapy (CBT)** | [35,39,50,51,53–56,62,65] | 10 | 25.58% |
| **Capability, Opportunity, Motivation-Behavior model (COM-B)** | [20,64,67] | 3 | 6.98% |
| **Motivational Interviewing (MI)** | [59,88] | 2 | 4.65% |
| Habit Formation Model (HFM) | [44,58] | 2 | 4.65% |
| Mohr’s Model of Supportive Accountability | [34] | 1 | 2.33% |
| Emotion regulation theory | [42] | 1 | 2.33% |
| 5A clinic practice guideline | [19] | 1 | 2.33% |
| ***Integrated Theory (8/43, 18.60%)*** |  |  |  |
| **CBT** and Positive Psychology (strength and resilience) | [52] | 1 | 2.33% |
| **CBT**, **MI**, and Emotionally Focused Therapy | [41] | 1 | 2.33% |
| **CBT and MI** | [46] | 1 | 2.33% |
| **COM-B** and the Theoretical Domains Framework | [57] | 1 | 2.33% |
| **MI** and Graded Exercise Therapy | [68] | 1 | 2.33% |
| **MI,** Behavioral Activation, Acceptance and Commitment Therapy, and Solution-Focused Brief Therapy | [16] | 1 | 2.33% |
| Chronic-Disease Extended Model extending from Health Belief Model, Theory of Planned Behavior, Diffusion of Innovation Theory, Social Norms Theory, Transtheoretical Model | [36] | 1 | 2.33% |
| Elaboration Likelihood Model, the Self-Regulatory Model of Message Framing, the Regulatory Focus Theory, and Theories of Emotions | [37] | 1 | 2.33% |
| Total | - | 28 | 65.12% |
| NR | - | 15 | 34.88% |

***Note.*** NR: not reported.

**Table S9.** Techniques for developing AI chatbots (n=43) [16–20,23,24,33–68]

| **Backend** | **Articles** | **Frontend** | **External service** | |
| --- | --- | --- | --- | --- |
| ***Non-code Platform (23/43)*** | | | | |
| Google Dialogflow | [17,20,35,40,57,63,64,66] | Facebook Messenger [17,20,35,63,66], Alexa [40], Mindbehind platform [57], LINE platform [64] | Google Cloud Functions and database [35], PostgreSQL [40], SlimMe DB [64], MongoDB & Firebase [17,63], Wearable device [20,35] | |
| IBM Watson | [16,24,42,44,48,49,58,65] | Facebook Messenger [42], Slack [48,49], Telegram [65], Short Message Service (SMS) [16], Text-based interface of mobile phone [24], FriendPlus [44,58] | MongoDB [16,42], Wearable device [48,49] | |
| Widebot | [36] | WhatsApp | N.R. | |
| Chatfuel | [23,67] | Facebook Messenger | N.R. | |
| Landbot | [61] | Web-interface | N.R. | |
| Rasa | [47] | Web-interface | N.R. | |
| X2AI (rebrand: Cass) | [39,41] | SMS or Facebook Messenger | N.R. | |
| ***Self-designed algorithm (8/43)*** | | | | |
| spaCy (NLP framework) | [31] | N.R. | N.R. | |
| NLP & Bayes’ theorem | [19] | Telegram | N.R. | |
| NLP & GPT-2 XL neural network | [23] | Web-interface | Google Firebase | |
| Supervised goal-based model | [38] | SMS | Wearable device | |
| NLP & Generative model & Dialogue management model | [68] | Telegram | N.R. | |
| Reinforcement learning-based algorithms | [43] | N.R. | N.R. | |
| Self-learning algorithm | [46] | N.R. | Google Fit (Google, Mountain View, California, USA) | |
| Microsoft Bot Framework | [33] | Inspire Support Hub | MySQL database | |
| ***Standalone App (12/43)*** | | | | |
| **App** | **Articles** | **Description** | **External Service** | |
| Woebot | [53–56,62] | Woebot helps users develop emotional regulation skills and supports mood monitoring and management through conversations. | N.R. | |
| Wysa | [18] | Wysa is not only an AI chatbot but also provides additional features, such as access to a human talk therapist, a journal for gratitude, and an SOS feature to seek help. | N.R. | |
| Vitalk | [52] | Vitalk is an AI-powered digital assistant for mental health. | N.R. | |
| Smoke-Free app | [34] | Smoke-Free app had a supportive AI chatbot for quitting smoking. | N.R. |  |
| CureApp Smoking Cessation (CASC) | [45] | CASC was designed to aid patients who were receiving outpatient smoking cessation treatment. | N.R. |  |
| Mobile personal health care agent (m-PHA) | [50,51] | m-PHA was designed to facilitate ABC (Activation, Belief, and Consequence) note writing for users. | N.R. |  |
| PsyMe app | [37] | PsyMe app was used for delivering persuasive messages (gain message, non-loss message, non-gain message, loss message) for promoting physical activity. | Wearable device |  |

***Note.*** N.R.: Not reported

**Table S10.** Efficacy and effectiveness of AI chatbot on health behavior change (n=33) [18–20,23,24,34–39,41–43,45–58,62,66–68,88]

| **Measurements** | **Study** | **Description** | **pre/CG** | | | **Post/IG** | | | **Post/IG vs Pre/CG** | |
| --- | --- | --- | --- | --- | --- | --- | --- | --- | --- | --- |
|  |  |  | M1/% | SD1/n1 | N1 | M2/% | SD2/n2 | N2 | Std. Diff with 95% CI (Hedges' g/ORs) | p-value |
| **Physical activity; 29/120: 24P (11 MCID), 4N (1 MCID), 1UD** | | | | | | | | | | |
| Moderate-to-vigorous PA (MVPA) minutes (minutes/week) | Maher et al. (2020) [48][Duration] | Pre vs. 6-week mid (↑) | 206.10 | 166.50 | 31 | 266.80 | 207.20 | 31 | 0.32 (-0.18, 0.82) | 0.212 |
|  |  | Pre vs. 12-week post (↑) | 206.10 | 166.50 | 31 | 315.90 | 261.70 | 31 | 0.49 (-0.01, 1.00) | 0.055 |
|  | Davis et al. (2020) [49][Engagement] | High engager: Pre vs. 12-week post (↑) | 272.00 | 206.00 | 13 | 418.00 | 227.00 | 13 | 0.65 (-0.14, 1.44) | 0.105 |
|  |  | Low engager: Pre vs.12-week post (↑) | 159.00 | 115.00 | 18 | 258.00 | 240.00 | 15 | 0.53 (-0.17, 1.23) | 0.136 |
|  | To et al. (2021) [20] | Pre vs. 6-week post (↑*) | 86.50 | 137.50 | 116 | 240.70 | 233.60 | 116 | 0.80 (0.53, 1.07) | 0.000 |
|  | Dhinagaran et al. (2021) [67] | Pre vs. 4-week post (↑) | 35.00 | 68.08 | 60 | 48.00 | 68.08 | 56 | 0.19 (-0.18, 0.56) | 0.308 |
| Steps (steps/day) | Hassoon et al. (2021) [38][RCTs] [Modalities] | CG: pre vs. 4-week post (↑) | 4847.00 | 2925.70 | 14 | 5593.70 | 4731.80 | 14 | 0.18 (-0.56, 0.93) | 0.627 |
|  |  | SmartText (Text): pre vs. 4-week post (↑) | 5522.40 | 3528.30 | 14 | 6924.80 | 4181.30 | 14 | 0.35 (-0.39, 1.10) | 0.355 |
|  |  | MyCoach (Audio): pre vs. 4-week post (↑*) | 5683.80 | 3194.20 | 14 | 9268.80 | 2895.20 | 14 | 1.14 (0.34, 1.94) | 0.005 |
|  | To et al. (2021) [20] | Pre vs. 6-week post (↑*) | 5933.00 | 2391.00 | 116 | 6570.00 | 2326.00 | 116 | 0.27 (0.01, 0.53) | 0.041 |
| % People participating in PA for at least one week | Albino De Queiroz et al. (2023) [35] | Pre vs. 8-week post (↑) | 38.46% | 5 | 13 | 100.00% | 13 | 13 | 3.73 (0.71, 6.75) | 0.015 |
| MET score/week | Dhinagaran et al. (2021) [67] | Pre vs. 4-week post (↓) | 1080.00 | 816.00 | 60 | 1075.00 | 872.00 | 56 | -0.01 (-0.37, 0.36) | 0.975 |
| % People increase in MET score | Carrasco-Hernandez et al. (2020) [46][RCTs] [Duration][Modalities] | 24-week mid: CG: traditional therapy vs. IG: traditional therapy and digital therapeutic solution (↓) | 14.60% | 6 | 45 | 11.76% | 6 | 51 | -0.14 (-1.35, 1.07) | 0.817 |
|  |  | 48-week post: CG vs. IG (↑) | 24.40% | 11 | 45 | 25.50% | 13 | 51 | 0.06 (-0.87, 0.98) | 0.906 |
| % Meet PA guideline (150 minutes of MVPA/week) | To et al. (2021) [20] | Pre vs. 6-week post (↑*) | 16.00 | 13.80 | 116 | 62.00 | 53.50 | 116 | 1.17 (0.89, 1.45) | 0.000 |
| Habit formation score on PA | Piao, Ryu et al. (2020) [58][RCTs] [Modalities] | Week 1 to 4: CG: chatbot with cues and extrinsic rewards vs. IG: chatbot with cues, intrinsic and extrinsic rewards (↑*) | 6.42 | 9.42 | 49 | 13.54 | 14.99 | 57 | 0.56 (0.17, 0.94) | 0.005 |
|  |  | Week 5 to 12: IG and CG received the same interventions: chatbot with cues, intrinsic and extrinsic rewards (↓) | 15.88 | 13.29 | 49 | 12.08 | 10.87 | 57 | -0.31 (-0.70, 0.07) | 0.110 |
| Self-efficacy | Albers, Hizli, et al. (2023) [47] [RCTs] [Modalities] | Pre vs. single session post (↓*) | Mean diff. = -12.38 | | 39 | SD diff. = 24.54 | | 39 | -0.49 (-0.83, -0.16) | 0.003 |
|  |  | CG: generic vs. IG: personalized (↓*) | Mean diff. = -3.57 | | 39 | SD diff. = 1.36 | | 39 | -2.57 (-3.22, -1.92) | 0.000 |
| Motivation |  | CG: generic vs. IG: personalized (↑*) | Mean diff. = 0.31 | | 39 | SD diff. = 1.19 | | 39 | 0.98 (0.60, 1.36) | 0.000 |
| Intention | Catellani et al. (2023) [37] [RCTs] [Engagement] | 2-week post (UD) | Higher message involvement positively affects the intention to participate in physical activity through feeling calmer (*β* = 0.07, *P* = 0.003) and experiencing more hope (*β* = 0.44, *P* < 0.001). | | | | | | | |
| % People enhanced motivation | Moore et al. (2024) [57] | Pre vs. single session post (↑) | 33.00% | 15 | 46 | 73.00% | 34 | 46 | 1.77 (0.87, 2.67) | 0.000 |
| % People enhanced confidence |  | Pre vs. single session post (↑) | 50.00% | 23 | 46 | 80.00% | 37 | 46 | 1.41 (0.48, 2.34) | 0.003 |
| Belief in PA engagement | Sun et al. (2023) [68] | CG: no vs. IG: stress topic (↓) | 3.40 | 0.69 | 30 | 2.97 | 0.96 | 30 | -0.51 (-1.02, 0.01) | 0.053 |
|  |  | CG: no vs. IG: fatigue (↑) | 2.90 | 1.04 | 31 | 3.27 | 0.83 | 31 | 0.39 (-0.11, 0.89) | 0.130 |
|  |  | CG: no vs. IG: motivation (↑) | 2.95 | 0.91 | 31 | 3.44 | 0.99 | 31 | 0.51 (0.00, 1.01) | 0.049 |
|  |  | CG: no vs. IG: time management (↑) | 3.23 | 0.85 | 28 | 3.40 | 0.78 | 28 | 0.21 (-0.32, 0.73) | 0.443 |
|  |  | CG: no vs. IG: habit formation (↑) | 3.28 | 0.72 | 32 | 3.57 | 0.44 | 32 | 0.48 (-0.02, 0.98) | 0.058 |
|  |  | CG: no vs. unsupportive social environment (↑) | 3.02 | 0.97 | 32 | 3.08 | 0.86 | 32 | 0.06 (-0.43, 0.55) | 0.796 |
| **Sedentary; 1/120: 1P (0 MCID)** | | | | | | | | | | |
| Sitting time (minutes/day) | Dhinagaran et al. (2021) [67] | Pre vs. 4-week post (↑) | 439.00 | N.R. | 60 | 407.00 | N.R. | 56 | Mean diff. = -32 | N.A. |
| **Diet; 14/120: 13P (5 MCID), 1UD** | | | | | | | | | | |
| Diet adherence | Davis et al. (2020) [49][Engagement] | High engager: Pre vs. 12-week post (↑*) | 3.80 | 2.00 | 13 | 10.80 | 3.10 | 13 | 2.60 (1.55, 3.64) | 0.000 |
|  |  | Low engager: Pre vs. 12-week post (↑*) | 3.80 | 1.70 | 18 | 9.70 | 1.40 | 15 | 3.66 (2.54, 4.78) | 0.000 |
|  | Maher et al. (2020) [48][Duration] | Pre vs. 6-week mid (↑*) | 3.80 | 1.80 | 31 | 9.80 | 3.70 | 31 | 2.04 (1.42, 2.65) | 0.000 |
|  |  | Pre vs. 12-week post (↑*) | 3.80 | 1.80 | 31 | 9.60 | 3.10 | 31 | 2.26 (1.62, 2.90) | 0.000 |
| Increase food consumption | Albino De Queiroz et al. (2023) [35] | 8-week post (UD) | 15.4% (2/13) of patients reported an increase in food consumption. | | | | | | | |
| % Consume vegetables at least once a day | Dhinagaran et al. (2021) [67] | Pre vs. 4-week post (↑) | 27.00% | 16 | 60 | 29.00% | 16 | 56 | 0.10 (-0.72, 0.91) | 0.819 |
| % Having three portions of fruit per day |  | Pre vs. 4-week post (↑) | 3.00% | 2 | 60 | 7.00% | 4 | 56 | 0.80 (-0.94, 2.54) | 0.366 |
| % Almost never consume sweetened beverages |  | Pre vs. 4-week post (↑) | 38.00% | 23 | 60 | 45.00% | 25 | 56 | 0.26 (-0.48, 1.00) | 0.491 |
| % Almost never consume fried food and snacks |  | Pre vs. 4-week post (↑) | 25.00% | 15 | 60 | 30.00% | 17 | 56 | 0.27 (-0.55, 1.08) | 0.519 |
| Gluten-free eating habits | Alghamdi & Alnanih (2021) [36][RCTs] | 12-week post (↑*) | The intervention group adopted significantly more gluten-free eating habits than control group at the end of the intervention (*F* = 50.62, *P* < 0.05) | | | | | | | |
| Nonclinical eating disorder | Fitzsimmons‐Craft et al. (2022) [39][RCTs] [Long-term] | Group x time (↑*) | 12-week: *β* = -0.12, *P* = 0.27, *d* = -0.1; 24-week: *β* = -0.33, *P* = 0.003, *d* = -0.29 | | | | | | | |
|  |  | Baseline: CG vs. IG (↑) | 3.50 | 1.07 | 348 | 3.38 | 1.04 | 352 | -0.11 (-0.26, 0.03) | 0.13 |
|  |  | 12-week follow-up: CG vs. IG (↑*) | 3.26 | 1.25 | 190 | 2.73 | 1.31 | 161 | -0.41 (-0.63, -0.20) | 0.00 |
|  |  | 24-week follow-up: CG vs. IG (↑) | 3.03 | 1.24 | 193 | 2.77 | 1.34 | 159 | -0.20 (-0.41, 0.01) | 0.06 |
| **Stress; 44/120: 35P (16 MCID), 4N (0 MCID), 5UD** | | | | | | | | | | |
| Perceived stress | Danieli et al. (2021) [50][Long-term] [RCTs][Modalities] | Traditional therapy: pre vs. 8-week post (↑) | 20.40 | 6.83 | 8 | 14.80 | 5.45 | 8 | -0.86 (-1.88, 0.17) | 0.101 |
|  |  | Traditional therapy: pre vs. 12-week follow-up (↑*) | 20.40 | 6.83 | 8 | 10.29 | 6.63 | 8 | -1.42 (-2.52, -0.32) | 0.011 |
|  |  | Multicomponent intervention: pre vs. 8-week post (↑*) | 22.09 | 2.21 | 11 | 16.55 | 5.45 | 11 | -1.28 (-2.20, -0.36) | 0.006 |
|  |  | Multicomponent intervention: pre vs. 12-week follow-up (↑) | 22.09 | 2.21 | 11 | 18.00 | 7.32 | 11 | -0.73 (-1.59, 0.14) | 0.098 |
|  | Danieli et al. (2022) [51][RCTs][Long-term] [Modalities] | Traditional therapy: pre vs. 8-week post (↑*) | 21.17 | 6.24 | 15 | 15.58 | 7.81 | 15 | -0.77 (-1.51, -0.03) | 0.042 |
|  |  | Traditional therapy: pre vs. 12-week follow-up (↑) | 21.17 | 6.24 | 15 | 16.92 | 5.45 | 15 | -0.71 (-1.44, 0.03) | 0.061 |
|  |  | Multicomponent intervention: pre vs. 8-week post (↑*) | 22.40 | 5.66 | 12 | 11.60 | 5.85 | 12 | -1.81 (-2.76, -0.86) | 0.000 |
|  |  | Multicomponent intervention: pre vs. 12-week follow-up (↑*) | 22.40 | 5.66 | 12 | 16.60 | 6.29 | 12 | -0.94 (-1.78, -0.09) | 0.030 |
|  |  | Chatbot only: pre vs. 8-week post (↑) | 21.50 | 8.17 | 8 | 14.00 | 9.38 | 8 | -0.81 (-1.83, 0.21) | 0.121 |
|  |  | Chatbot only: pre vs. 12-week follow-up (↑) | 21.50 | 8.17 | 8 | 18.67 | 7.53 | 8 | -0.34 (-1.33, 0.65) | 0.499 |
|  |  | Control: pre vs. 8-week post (↓) | 13.87 | 5.19 | 10 | 14.63 | 7.15 | 10 | 0.12 (-0.76, 0.99) | 0.795 |
|  |  | Control: pre vs. 12-week follow-up (↓) | 13.87 | 5.19 | 10 | 15.13 | 5.25 | 10 | 0.23 (-0.65, 1.11) | 0.606 |
|  | Karhiy et al. (2023) [24][RCTs] [Modalities] | Digital human: pre vs. 4-week post (↑) | All groups had significantly reduced perceived stress at follow-up compared to baseline (*F*(1,100) = 23.37, *P* < 0.001, η2 = 0.19). However, there was no significant interaction between group and time. | | | | | | d = 0.52 | N.A. |
|  |  | Teletherapy: pre vs. 4-week post (↑) |  |  |  |  |  |  | d = 0.54 | N.A. |
|  |  | Chatbot: pre vs. 4-week post (↑) |  |  |  |  |  |  | d = 0.36 | N.A. |
|  | Meng & Dai (2021) [23][RCTs][Engagement] | One-time: emotional support & stress reduction (UD) | Emotional support did not have a significant effect on stress reduction (*F*(1, 169) = 1.06, *P* = 0.31, η2 = 0.01). However, receiving emotional support increased a discloser’s perceived supportiveness (*β* = 0.99, *P* < 0.001), which in turn, predicted stress reduction (*β* = 0.13, *P* = 0.003). | | | | | | | |
|  | Dhinagaran et al. (2021) [67] | Pre vs. 4-week post (↑) | 17.00 | 5.13 | 60 | 16.00 | 5.10 | 56 | -0.19 (-0.56, 0.17) | 0.297 |
|  | Legaspi Jr. et al. (2022) [18] | Pre vs. 1-week post (↑) | N.R. | | | | | | Mean diff. = -8.3 | N.A. |
|  | Sun et al. (2023) [68] | 3 vs. single session post (↑*) | 3.00 | N.A. | N.A. | 3.60 | 0.72 | 30 | *t*_29_ = 4.52 | 0.001 |
|  | De Nieva et al., 2020) [53] | Pre vs. 2-week post (↑) | 23.72 | N.R. | 25 | 19.48 | N.R. | 25 | Mean diff. = -4.24 | N.A. |
|  | Forman-Hoffman et al. (2023) [55] [Effectiveness] | Location difference & stress reduction (UD) | Change in stress at week 8: Non-MUA vs. MUA (*t*_253_ = 0.30, *P* = 0.77, d = 0.04 95% CI [-0.23, 0.30]); Non-MHPSA vs. MHPSA (*t*_253_ = -1.39, *P* = 0.17, d = -0.18 95% CI [-0.44, 0.07]) | | | | | | | |
|  |  | Non-MUA: pre vs. 8-week post (↑*) | 21.29 | 5.04 | 156 | 18.84 | 5.87 | 156 | -0.45 (-0.67, -0.22) | 0.000 |
|  |  | MUA: pre vs. 8-week post (↑*) | 21.38 | 6.41 | 99 | 18.58 | 5.97 | 99 | -0.45 (-0.73, -0.17) | 0.002 |
|  |  | Non-MHPSA: pre vs. 8-week post (↑*) | 22.13 | 4.88 | 114 | 19.04 | 5.90 | 114 | -0.57 (-0.83, -0.30) | 0.000 |
|  |  | MHPSA: pre vs. 8-week post (↑*) | 20.67 | 6.06 | 141 | 18.48 | 5.90 | 141 | -0.37 (-0.60, -0.13) | 0.002 |
|  | Hoffman et al. (2023) [56] [Effectiveness] | Group x Time (UD) | *F*(2, 119.52) = 3.54, *P* = 0.03 | | | | | | | |
|  |  | Typical utilizer: pre vs. 8-week post (↑*) | Mean diff. = -1.4 | | 81 | SD diff. = 5.5 | | 81 | -0.25 (-0.47, -0.03) | 0.025 |
|  |  | Early utilizer: pre vs. 8-week post (↑*) | Mean diff. = -3.1 | | 58 | SD diff. = 7 | | 58 | -0.44 (-0.71, -0.17) | 0.001 |
|  |  | Efficient engager: pre vs. 8-week post (↑*) | Mean diff. = -4.1 | | 63 | SD diff. = 6.8 | | 63 | -0.60 (-0.86, -0.33) | 0.000 |
|  | Durden et al. (2023) [54][Effectiveness] | Total population: pre vs. 8-week post (↑*) | 21.36 | 5.61 | 256 | 18.71 | 5.90 | 256 | -0.46 (-0.64, -0.28) | 0.000 |
|  |  | Non-clinically elevated mood symptoms: pre vs. 8-week post (↑*) | 18.68 | 5.13 | 117 | 17.10 | 6.19 | 117 | -0.28 (-0.53, -0.02) | 0.035 |
|  |  | Clinically elevated mood symptoms: pre vs. 8-week post (↑*) | 23.60 | 4.99 | 139 | 20.07 | 5.30 | 139 | -0.68 (-0.93, -0.44) | 0.000 |
|  | Daley et al. (2020) [52][Effectiveness][Engagement] | Pre vs. 4-week post (↑*) | 25.80 | 9.10 | 1648 | 17.50 | 9.30 | 1648 | -0.90 (-0.97, -0.83) | 0.000 |
|  |  | Engagement & stress reduction (UD) | Increase in engagement did not significantly decrease stress (*β* = 0.11, *P* = 0.716) | | | | | | | |
| Current stress (low stress: high arousal and low valence) | Medeiros et al. (2022) [42] [RCTs] [Modalities] | Bot_arousal: Pre vs. 3-day post (↓) | Mean diff. = -0.02 | | 32 | SD diff. = 1.08 | | 32 | -0.02 (-0.36, 0.33) | 0.917 |
|  |  | Human_arousal: Pre vs. 3-day post (↑) | Mean diff. = 0.21 | | 24 | SD diff. = 1.38 | | 24 | 0.15 (-0.26, 0.55) | 0.464 |
|  |  | Control_arousal: Pre vs. 3-day post (↑) | Mean diff. = 0.03 | | 30 | SD diff. = 1.33 | | 30 | 0.02 (-0.34, 0.38) | 0.903 |
|  |  | Bot_valence: Pre vs. 3-day post (↑) | Mean diff. = -0.18 | | 32 | SD diff. = 0.85 | | 32 | -0.21 (-0.56, 0.14) | 0.240 |
|  |  | Human_valence: Pre vs. 3-day post (↑*) | Mean diff. = -1.1 | | 24 | SD diff. = 1.63 | | 24 | -0.65 (-1.09, -0.21) | 0.003 |
|  |  | Control_valence: Pre vs. 3-day post (↑) | Mean diff. = -0.18 | | 30 | SD diff. = 0.85 | | 30 | -0.21 (-0.57, 0.16) | 0.256 |
|  | Karhiy et al. (2023) [24][RCTs] [Modalities] | 3 Groups x time (↑*) | There was a significant decrease in current stress over time in all groups (*F*(2,200) = 59.90, *P* < 0.001, η2 = 0.38) | | | | | | | |
| Heart Rate Variability |  | 3 Groups x time (UD) | *P* > 0.05 | | | | | | | |
| Skin temperature |  | 3 Groups x time (↑*) | There was a significant increase in skin temperature over time in all groups (*F*(1,100) = 127.59, *P* < 0.001, η2 = 0.56) | | | | | | | |
| Electrodermal activity |  | 3 Groups x time (↓*) | There was a significant increase in electrodermal activity over time in all groups (*F*(2,100) = 6.21, *P* = -0.0003, η = 0.11) | | | | | | | |
| **Sleep; 3/120: 1P (0 MCID), 2N (0 MCID)** | | | | | | | | | | |
| Sleep quality | Dhinagaran et al. (2021)[67] | Pre vs. 4-week post (↓) (PSQI >5: poor sleep quality) | 4.38 | 2.36 | 60 | 4.43 | 2.45 | 56 | 0.02 (-0.34, 0.38) | 0.911 |
|  | Sia et al. (2021) [66] | Pre vs. 1-week post (↑) (large score: good quality) | 2.28 | N.R. | 36 | 2.40 | N.R. | 36 | Mean diff. = 0.12 | N.A. |
| % Sleeping for less than 7 hours | Dhinagaran et al. (2021) [67] | Pre vs. 4-week post (↓) | 0.82 | N.R. | 60 | 0.88 | N.R. | 56 | Mean diff. = 0.06 | N.A. |
| **Weight management; 8/120: 6P (0 MCID), 1N (0 MCID), 1UD** | | | | | | | | | | |
| Body mass index (BMI) | Carrasco-Hernandez et al. (2020) [46] [RCTs] [Duration] [Modalities] | 24-week mid: CG vs. IG (↑) | 1.10 | 1.38 | 45 | 1.01 | 1.92 | 51 | -0.05 (-0.45, 0.35) | 0.796 |
|  |  | 48-week post: CG vs. IG (↓) | 1.22 | 1.85 | 45 | 1.47 | 2.06 | 51 | 0.13 (-0.28, 0.53) | 0.537 |
|  | To et al. (2021) [20] | Pre vs. 6-week post (↑) | 32.50 | 8.00 | 116 | 32.40 | 8.00 | 116 | -0.01 (-0.27, 0.24) | 0.924 |
| Body weight (kg) | Maher et al. (2020) [48][Duration] | Pre vs. 6-week mid (↑) | 83.60 | 19.00 | 31 | 82.40 | 18.30 | 31 | -0.06 (-0.56, 0.43) | 0.803 |
|  |  | Pre vs. 12-week post (↑) | 83.60 | 19.00 | 31 | 82.30 | 18.10 | 31 | -0.07 (-0.57, 0.43) | 0.785 |
| Waist circumference (cm) |  | Pre vs. 6-week mid (↑) | 96.00 | 16.00 | 31 | 95.10 | 15.80 | 31 | -0.06 (-0.55, 0.44) | 0.826 |
|  |  | Pre vs. 12-week post (↑) | 96.00 | 16.00 | 31 | 93.90 | 15.80 | 31 | -0.13 (-0.63, 0.37) | 0.608 |
| Target goal progress for weight management | Stephens et al. (2019) [41] | 10–12-week post (UD) | Adolescent patients reported experiencing positive progress toward their goals and targeted behaviors 81% of the time. | | | | | | | |
| **Smoking; 20/120: 17P (11 MCID), 1N (0 MCID), 2UD** | | | | | | | | | | |
| Biochemically validated abstinence rate | Carrasco-Hernandez et al. (2020) [46][RCTs] [Duration][Modalities] | CG vs. IG (per-protocol) (↑*) | 40.00% | 18 | 45 | 64.70% | 33 | 51 | 1.01 (0.18, 1.84) | 0.017 |
|  | Olano-Espinosa et al. (2022) [19][RCTs] [Duration][Engagement] | CG: usual care by human vs. IG: chatbot (per-protocol) (↑*) | 39.80% | 51 | 128 | 60.60% | 63 | 104 | 0.84 (0.31, 1.37) | 0.002 |
|  |  | IG: non-intensive vs Intensive (>4 contacts and > 30 of total interaction time) (↑*) | 40.90% | 43 | 104 | 68.60% | 71 | 104 | 1.12 (0.55, 1.68) | 0.000 |
|  |  | CG: non-intensive vs. Intensive (↑*) | 35.40% | 45 | 128 | 47.60% | 61 | 128 | 0.52 (0.02, 1.02) | 0.043 |
|  | Masaki et al. (2019) [45] [Long-term] | 9-week mid vs. 12-week post (↑*) | Mean diff. = 0.67 | | 54 | SD diff. = 0.47 | | 54 | 1.40 (1.03, 1.78) | 0.000 |
|  |  | 9-week vs. 24-week follow-up (↑*) | Mean diff. = 0.76 | | 53 | SD diff. = 0.43 | | 53 | 1.74 (1.31, 2.17) | 0.000 |
|  |  | 9-week vs. 52-week follow-up (↑*) | Mean diff. = 0.58 | | 51 | SD diff. = 0.46 | | 51 | 1.24 (0.88, 1.61) | 0.000 |
| % Quit success | Perski et al. (2019) [34][RCTs] | CG: ‘pro’ app vs. IG: ‘pro’ app & AI chatbot (↑*) | 73.35% | 3704 | 5050 | 79.55% | 844 | 1061 | 0.35 (0.18, 0.51) | 0.000 |
| % Quit attempts | Brown et al. (2023) [88] [Long-term] | Pre vs. single session post (↓) | 40.00% | 40 | 100 | 40.00% | 38 | 100 | -0.08 (-0.65, 0.48) | 0.772 |
| % Number of quits |  | Single session post (UD) | NR | | | 74.00% | 74 | 100 | N.A. | N.A. |
| Confidence in quitting smoking |  | Pre vs. single session post (↑*) | 3.30 | 2.30 | 100 | 4.10 | 2.50 | 100 | 0.33 (0.05, 0.61) | 0.020 |
|  |  | Pre vs. 1-week follow-up (↑*) | 3.30 | 2.30 | 100 | 4.70 | 2.70 | 100 | 0.56 (0.27, 0.84) | 0.000 |
| Importance of quitting smoking |  | Pre vs. single session post (↑) | 5.50 | 2.90 | 100 | 6.00 | 2.80 | 100 | 0.17 (-0.10, 0.45) | 0.217 |
|  |  | Pre vs. 1-week follow-up (↑) | 5.50 | 2.90 | 100 | 6.20 | 2.80 | 100 | 0.24 (-0.03, 0.52) | 0.085 |
| Readiness to quit smoking |  | Pre vs. single session post (↑) | 4.90 | 2.80 | 100 | 5.30 | 2.70 | 100 | 0.14 (-0.13, 0.42) | 0.306 |
|  |  | Pre vs. 1-week follow-up (↑) | 4.90 | 2.80 | 100 | 5.40 | 2.90 | 100 | 0.17 (-0.10, 0.45) | 0.217 |
| Tobacco cravings | Masaki et al. (2019) [45] [Long-term] | Pre vs. 24-week post (↑*) | 3.20 | 0.70 | 53 | 2.00 | 1.10 | 53 | -1.29 (-1.71, -0.87) | 0.000 |
| Social nicotine dependence |  | Pre vs. 24-week post (↑*) | 16.60 | 4.30 | 53 | 10.50 | 5.90 | 53 | -1.17 (-1.59, -0.76) | 0.000 |
| Severity of withdrawal symptoms |  | Pre vs. 24-week post (↑*) | 20.60 | 4.70 | 53 | 13.70 | 3.50 | 53 | -1.65 (-2.09, -1.21) | 0.000 |
| Ease and motivation to quit smoking | Albers, Neerincx et al. (2023) [43] | 9-day post (UD) | Positive association between willingness to continue and their ease of 0.33 (95% HDI = [0.25, 0.41]) and motivation 0.48 (95% HDI = [0.40, 0.54]) to quit smoking. | | | | | | | |
| **Alcohol; 1/120: 1 P (0 MCID)** | | | | | | | | | | |
| Alcohol use disorders | Prochaska et al. (2021) [62] | Pre vs. 8-week post (↑*) | 5.30 | 2.90 | 51 | 4.00 | 3.20 | 51 | -0.42 (-0.81, -0.03) | 0.035 |

**P (Positive change):** Refers to improvements such as increased physical activity, healthier eating, better sleep quality and quantity, reduced sedentary behavior, lower stress, weight reduction, or decreased smoking and alcohol use. Specifically, **↑** indicates a non-significant positive change, while **↑*** denotes a statistically significant positive change.

**N (Negative change):** Refers to outcomes opposite to P. Specifically, **↓** indicates a non-significant negative change, while **↓*** denotes a statistically significant negative change.

**UD (Unable to determine):** Used when the outcome direction cannot be identified.

**MCID (Minimal clinically important difference):** The minimal detectable health behavior change was set at g/OR/d > 0.5.

**g (Hedges’ g):** An effect size calculated from the mean and standard deviation of pre–post or control–intervention comparisons;

**OR (Odds Ratio):** An effect size calculated from the number of events in pre–post or control–intervention comparisons;

**d (Cohen's d):** An [effect size](https://www.google.com/search?q=effect+size&rlz=1C1CHBF_enUS1127US1127&oq=d+%28Cohen%27s+d%29%3A&gs_lcrp=EgZjaHJvbWUyBggAEEUYOTIKCAEQABgKGBYYHjIKCAIQABgKGBYYHjIICAMQABgWGB4yCAgEEAAYFhgeMggIBRAAGBYYHjIKCAYQABgKGBYYHjIGCAcQRRhB0gEHNTQ3ajBqNKgCA7ACAfEFLqeBnku58pw&sourceid=chrome&ie=UTF-8&mstk=AUtExfCiHTXsVr6ZitOKL6igfoTfKXIIVWvt6qzbgkv0vMn6vZJgYLXlN-nK-rWbcxZFfwPtxunSSkoQsrZu7PrK_4fWbYxhF7zpgOhxw1xZ7jij_rZocKUC_yJawIILi4Y-fEA_dC-lOQunI2Iks_0rPk94PhT8ddu0HPWRweCjbDlp2ow&csui=3&ved=2ahUKEwju0sfeprGQAxUVg4kEHdggIxUQgK4QegQIARAF) that quantifies the magnitude of the difference between two group means in units of standard deviation.

**MHPSA:** mental health provider shortage area; **MUA:** medically underserved area

**CI:** Confidence interval; **HDI:** Highest Density Interval

**N.A.:** Not available; **N.R.:** Not reported.

**References**

16. Figueroa CA, Luo TC, Jacobo A, Munoz A, Manuel M, Chan D, et al. Conversational physical activity coaches for Spanish and English speaking women: a user design study. Front Digit Health. 2021;3:747153. [FREE Full text] [doi:10.3389/fdgth.2021.747153] [Medline: 34713207]

17. Holmes S, Moorhead A, Bond R, Zheng H, Coates V, Mctear M. Usability testing of a healthcare chatbot: can we use conventional methods to assess conversational user interfaces? 2019. Presented at: Proceedings of the 31st European Conference on Cognitive Ergonomics; September 10-13, 2019:207-214; Belfast, United Kingdom. [doi: 10.1145/3335082.3335094]

18. Legaspi CM, Pacana TR, Loja K, Sing C, Ong E. User perception of Wysa as a mental well-being support tool during the COVID-19 pandemic. 2022. Presented at: Asian HCI Symposium ’22. ACM; April 29-May 5, 2022:52-57; New Orleans, LA, United States. [doi: 10.1145/3516492.3559064]

19. Olano-Espinosa E, Avila-Tomas JF, Minue-Lorenzo C, Matilla-Pardo B, Serrano Serrano ME, Martinez-Suberviola FJ, et al. Effectiveness of a conversational chatbot (Dejal@bot) for the adult population to quit smoking: pragmatic, multicenter, controlled, randomized clinical trial in primary care. JMIR Mhealth Uhealth. 2022;10(6):e34273. [FREE Full text] [doi: 10.2196/34273] [Medline: 35759328]

20. To QG, Green C, Vandelanotte C. Feasibility, usability, and effectiveness of a machine learning-based physical activity chatbot: quasi-experimental study. JMIR Mhealth Uhealth. 2021;9(11):e28577. [FREE Full text] [doi: 10.2196/28577] [Medline: 34842552]

23. Meng J, Dai YN. Emotional support from AI chatbots: should a supportive partner self-disclose or not? J Comput-Mediat Commun. 2021;26(4):207-222. [doi: 10.1093/jcmc/zmab005]

24. Karhiy M, Sagar M, Antoni M, Loveys K, Broadbent E. Mindfulness based stress reduction: a randomised trial of a virtual human, teletherapy, and a chatbot. 2023. Presented at: 2023 11th International Conference on Affective Computing and Intelligent Interaction Workshops and Demos (ACIIW); September 10-13, 2023:1-7; Cambridge, MA, United States. [doi: 10.1109/aciiw59127.2023.10388195]

33. Cameron G, Cameron D, Megaw G. Assessing the usability of a chatbot for mental health care. In: Lecture Notes in Computer Science. 2018. Presented at: INSCI 2018 International Workshop; October 24-26, 2018:121-132; St Petersburg, Russia. [doi: 10.1007/978-3-030-17705-8_11]

34. Perski O, Crane D, Beard E, Brown J. Does the addition of a supportive chatbot promote user engagement with a smoking cessation app? An experimental study. Digit Health. 2019;5:2055207619880676. [FREE Full text] [doi: 10.1177/2055207619880676] [Medline: 31620306]

35. Albino de Queiroz D, Silva Passarello R, Veloso de Moura Fé V, Rossini A, Folchini da Silveira E, Aparecida Isquierdo Fonseca de Queiroz E, et al. A wearable chatbot-based model for monitoring colorectal cancer patients in the active phase of treatment. Healthc Anal. 2023;4:100257. [doi: 10.1016/j.health.2023.100257]

36. Alghamdi E, Alnanih R. Chatbot design for a healthy life to celiac patients: a study according to a new behavior change model. Int J Adv Comput Sci Appl. 2021;12(10):698-707. [doi: 10.14569/ijacsa.2021.0121077]

37. Catellani P, Biella M, Carfora V, Nardone A, Brischigiaro L, Manera MR, et al. A theory-based and data-driven approach to promoting physical activity through message-based interventions. Front Psychol. 2023;14:1200304. [FREE Full text] [doi: 10.3389/fpsyg.2023.1200304] [Medline: 37575427]

38. Hassoon A, Baig Y, Naiman DQ, Celentano DD, Lansey D, Stearns V, et al. Randomized trial of two artificial intelligence coaching interventions to increase physical activity in cancer survivors. NPJ Digit Med. 2021;4(1):168. [FREE Full text] [doi: 10.1038/s41746-021-00539-9] [Medline: 34887491]

39. Fitzsimmons-Craft EE, Chan WW, Smith AC, Firebaugh M, Fowler LA, Topooco N, et al. Effectiveness of a chatbot for eating disorders prevention: a randomized clinical trial. Int J Eat Disord. 2022;55(3):343-353. [doi: 10.1002/eat.23662] [Medline: 35274362]

40. Larizza C, Bosoni P, Quaglini S, Chasseur M, Bevolo V, Zuccotti G, et al. V-care: an application to support lifestyle improvement in children with obesity. Int J Med Inform. 2023;177:105140. [doi: 10.1016/j.ijmedinf.2023.105140] [Medline: 37463558]

41. Stephens TN, Joerin A, Rauws M, Werk LN. Feasibility of pediatric obesity and prediabetes treatment support through Tess, the AI behavioral coaching chatbot. Transl Behav Med. 2019;9(3):440-447. [doi: 10.1093/tbm/ibz043] [Medline: 31094445]

42. Medeiros L, Bosse T, Gerritsen C. Can a chatbot comfort humans? Studying the impact of a supportive chatbot on users' self-perceived stress. IEEE Trans Human-Mach Syst. 2022;52(3):343-353. [doi: 10.1109/THMS.2021.3113643]

43. Albers N, Neerincx MA, Aretz NL, Ali M, Ekinci A, Brinkman WP. Attitudes toward a virtual smoking cessation coach: relationship and willingness to continue. In: Meschtscherjakov A, Midden C, Ham J, editors. Persuasive Technology. PERSUASIVE 2023. Lecture Notes in Computer Science, vol 13832. Switzerland. Springer; 2023:265-274.

44. Piao M, Kim J, Ryu H, Lee H. Development and usability evaluation of a healthy lifestyle coaching chatbot using a habit formation model. Healthc Inform Res. 2020;26(4):255-264. [FREE Full text] [doi: 10.4258/hir.2020.26.4.255] [Medline: 33190459]

45. Masaki K, Tateno H, Kameyama N, Morino E, Watanabe R, Sekine K, et al. Impact of a novel smartphone app (CureApp smoking cessation) on nicotine dependence: prospective single-arm interventional pilot study. JMIR Mhealth Uhealth. 2019;7(2):e12694. [FREE Full text] [doi: 10.2196/12694] [Medline: 30777848]

46. Carrasco-Hernandez L, Jódar-Sánchez F, Núñez-Benjumea F, Moreno Conde J, Mesa González M, Civit-Balcells A, et al. A mobile health solution complementing psychopharmacology-supported smoking cessation: randomized controlled trial. JMIR Mhealth Uhealth. 2020;8(4):e17530. [FREE Full text] [doi: 10.2196/17530] [Medline: 32338624]

47. Albers N, Hizli B, Scheltinga BL, Meijer E, Brinkman W-P. Setting physical activity goals with a virtual coach: Vicarious experiences, personalization and acceptance. J Med Syst. 2023;47(1):15. [FREE Full text] [doi: 10.1007/s10916-022-01899-9] [Medline: 36710276]

48. Maher CA, Davis CR, Curtis RG, Short CE, Murphy KJ. A physical activity and diet program delivered by artificially intelligent virtual health coach: proof-of-concept study. JMIR Mhealth Uhealth. 2020;8(7):e17558. [FREE Full text] [doi: 10.2196/17558] [Medline: 32673246]

49. Davis CR, Murphy KJ, Curtis RG, Maher CA. A process evaluation examining the performance, adherence, and acceptability of a physical activity and diet artificial intelligence virtual health assistant. Int J Environ Res Public Health. 2020;17(23):9137. [FREE Full text] [doi: 10.3390/ijerph17239137] [Medline: 33297456]

50. Danieli M, Ciulli T, Mousavi SM, Riccardi G. A conversational artificial intelligence agent for a mental health care app: evaluation study of its participatory design. JMIR Form Res. 2021;5(12):e30053. [FREE Full text] [doi: 10.2196/30053] [Medline: 34855607]

51. Danieli M, Ciulli T, Mousavi SM, Silvestri G, Barbato S, Di Natale L, et al. Assessing the impact of conversational artificial intelligence in the treatment of stress and anxiety in aging adults: randomized controlled trial. JMIR Ment Health. 2022;9(9):e38067. [FREE Full text] [doi: 10.2196/38067] [Medline: 36149730]

52. Daley K, Hungerbuehler I, Cavanagh K, Claro HG, Swinton PA, Kapps M. Preliminary evaluation of the engagement and effectiveness of a mental health chatbot. Front Digit Health. 2020;2:576361. [FREE Full text] [doi: 10.3389/fdgth.2020.576361] [Medline: 34713049]

53. De Nieva JO, Joaquin JA, Tan CB, Marc Te RK, Ong E. Investigating students’ use of a mental health chatbot to alleviate academic stress. 2021. Presented at: 6th International ACM In-Cooperation HCI and UX Conference; October 21-23, 2020:1-10; Jakarta & Bandung, Indonesia. [doi: 10.1145/3431656.3431657]

54. Durden E, Pirner MC, Rapoport SJ, Williams A, Robinson A, Forman-Hoffman VL. Changes in stress, burnout, and resilience associated with an 8-week intervention with relational agent "Woebot". Internet Interv. 2023;33:100637. [FREE Full text] [doi: 10.1016/j.invent.2023.100637] [Medline: 37635948]

55. Forman-Hoffman VL, Pirner MC, Flom M, Kirvin-Quamme A, Durden E, Kissinger JA, et al. Engagement, satisfaction, and mental health outcomes across different residential subgroup users of a digital mental health relational agent: exploratory single-arm study. JMIR Form Res. 2023;7:e46473. [FREE Full text] [doi: 10.2196/46473] [Medline: 37756047]

56. Hoffman V, Flom M, Mariano TY, Chiauzzi E, Williams A, Kirvin-Quamme A, et al. User engagement clusters of an 8-week digital mental health intervention guided by a relational agent (Woebot): exploratory study. J Med Internet Res. 2023;25:e47198. [FREE Full text] [doi: 10.2196/47198] [Medline: 37831490]

57. Moore R, Al-Tamimi A-K, Freeman E. Investigating the potential of a conversational agent (Phyllis) to support adolescent health and overcome barriers to physical activity: co-design study. JMIR Form Res. 2024;8:e51571. [FREE Full text] [doi: 10.2196/51571] [Medline: 38294857]

58. Piao M, Ryu H, Lee H, Kim J. Use of the healthy lifestyle coaching chatbot app to promote stair-climbing habits among office workers: exploratory randomized controlled trial. JMIR Mhealth Uhealth. 2020;8(5):e15085. [FREE Full text] [doi: 10.2196/15085] [Medline: 32427114]

59. Almusharraf F, Rose J, Selby P. Engaging unmotivated smokers to move toward quitting: design of motivational interviewing-based chatbot through iterative interactions. J Med Internet Res. 2020;22(11):e20251. [FREE Full text] [doi: 10.2196/20251] [Medline: 33141095]

60. Brown A, Kumar AT, Melamed O, Ahmed I, Wang YH, Deza A, et al. A motivational interviewing chatbot with generative reflections for increasing readiness to quit smoking: iterative development study. JMIR Ment Health. 2023;10:e49132. [FREE Full text] [doi: 10.2196/49132] [Medline: 37847539]

61. Aarts T, Markopoulos P, Giling L, Vacaretu T, Pillen S. Snoozy: a chatbot-based sleep diary for children aged eight to twelve. 2022. Presented at: IDC '22: Interaction Design and Children; June 27-30, 2022:297-307; Braga, Portugal. [doi: https://doi.org/10.1145/3501712.3529718]

62. Prochaska JJ, Vogel EA, Chieng A, Kendra M, Baiocchi M, Pajarito S, et al. A therapeutic relational agent for reducing problematic substance use (Woebot): development and usability study. J Med Internet Res. 2021;23(3):e24850. [FREE Full text] [doi: 10.2196/24850] [Medline: 33755028]

63. Griol D, Callejas Z, Fernandez-Martinez F, Esposito A. An application of conversational systems to promote healthy lifestyle habits. 2022. Presented at: 2022 IEEE Intl Conf on Dependable, Autonomic and Secure Computing, Intl Conf on Pervasive Intelligence and Computing, Intl Conf on Cloud and Big Data Computing, Intl Conf on Cyber Science and Technology Congress (DASC/PiCom/CBDCom/CyberSciTech); September 12-15, 2022:1-6; Falerna, Italy. [doi: 10.1109/dasc/picom/cbdcom/cy55231.2022.9927835]

64. Rahmanti AR, Yang H, Bintoro BS, Nursetyo AA, Muhtar MS, Syed-Abdul S, et al. SlimMe, a chatbot with artificial empathy for personal weight management: system design and finding. Front Nutr. 2022;9:870775. [FREE Full text] [doi: 10.3389/fnut.2022.870775] [Medline: 35811989]

65. Fadhil A, AbuRa’ed A. OlloBot—towards a text-based Arabic health conversational agent: evaluation and results. 2019. Presented at: Proceedings—Natural Language Processing in a Deep Learning World; September 2-4, 2019:295-303; Shoumen, Bulgaria. [doi: 10.26615/978-954-452-056-4_034]

66. Sia DE, Yu MJ, Daliva JL, Montenegro J, Ong E. Investigating the acceptability and perceived effectiveness of a chatbot in helping students assess their well-being. 2021. Presented at: Asian CHI Symposium 2021; May 8-13, 2021:34-40; Yokohama, Japan. [doi: 10.1145/3429360.3468177]

67. Dhinagaran DA, Sathish T, Soong A, Theng Y, Best J, Tudor Car L. Conversational agent for healthy lifestyle behavior change: web-based feasibility study. JMIR Form Res. 2021;5(12):e27956. [FREE Full text] [doi: 10.2196/27956] [Medline: 34870611]

68. Sun X, Casula D, Navaratnam A. Virtual support for real-world movement: using chatbots to overcome barriers to physical activity. 2023. Presented at: 2nd International Conference on Hybrid Human-Artificial Intelligence; June 26-30, 2023:201-214; Munich, Germany. [doi: 10.3233/FAIA230084]
